# Retrospective analysis of the Italian exit strategy from COVID-19 lockdown

**DOI:** 10.1073/pnas.2019617118

**Published:** 2021-01-07

**Authors:** Valentina Marziano, Giorgio Guzzetta, Bruna Maria Rondinone, Fabio Boccuni, Flavia Riccardo, Antonino Bella, Piero Poletti, Filippo Trentini, Patrizio Pezzotti, Silvio Brusaferro, Giovanni Rezza, Sergio Iavicoli, Marco Ajelli, Stefano Merler

**Affiliations:** ^a^Center for Health Emergencies, Bruno Kessler Foundation, Trento 38123, Italy;; ^b^Department of Occupational and Environmental Medicine, Epidemiology and Hygiene, Italian Workers’ Compensation Authority, Monteporzio Catone (Rome) 00078, Italy;; ^c^Department of Infectious Diseases, Istituto Superiore di Sanità, Rome 00161, Italy;; ^d^Department of Epidemiology and Biostatistics, Indiana University School of Public Health, Bloomington, IN 47405;; ^e^Laboratory for the Modeling of Biological and Socio-technical Systems, Northeastern University, Boston, MA 02115

**Keywords:** SARS-CoV-2, reopening scenarios, mathematical modeling

## Abstract

After the national lockdown imposed on March 11, 2020, the Italian government has gradually resumed the suspended economic and social activities since May 4, while maintaining the closure of schools until September 14. We use a model of severe acute respiratory syndrome coronavirus 2 (SARS-CoV-2) transmission to estimate the health impact of different exit strategies. The strategy adopted in Italy kept the reproduction number R_t_ at values close to one until the end of September, with marginal regional differences. Based on the estimated postlockdown transmissibility, reopening of workplaces in selected industrial activities might have had a minor impact on the transmissibility. Reopening educational levels in May up to secondary schools might have influenced SARS-CoV-2 transmissibility only marginally; however, including high schools might have resulted in a marked increase of the disease burden. Earlier reopening would have resulted in disproportionately higher hospitalization incidence. Given community contacts in September, we project a large second wave associated with school reopening in the fall.

## Significance

We use a mathematical model to evaluate the Italian exit strategy after the lockdown imposed against the COVID-19 epidemics, comparing it to a number of alternative scenarios. We highlight that a successful reopening requires two critical conditions: a low value of the reproduction number and a low incidence of infection. The first is needed to allow some margin for expansion after the lifting of restrictions; the second is needed because the level of incidence will be maintained approximately constant after the reproduction number has grown to values close to one. Furthermore, we suggest that, even with significant reductions of transmission rates, resuming social contacts at prepandemic levels escalates quickly the COVID-19 burden.

Since the declaration of the COVID-19 pandemic (1), a large number of countries worldwide have applied unprecedented restrictive measures to prevent the disease from overwhelming national health systems. The measures aimed at reducing the number of social contacts by enforcing physical distancing, and included different degrees of school closures, suspension of nonessential productive activities, stopping of mass gatherings and events, reduction of internal and international flights, and individual movement restrictions (2). Italy, the first country to experience a widespread epidemic in the western hemisphere, was also the first country outside of Asia to impose a generalized lockdown on March 11, 2020, allowing its citizens to leave their homes only in selected circumstances, that is, medical needs, grocery or pharmacy shopping, and commuting to work for essential jobs, with all of the others suspended or converted to smart working (3). These interventions have proven successful in curbing the spread of the disease (4–6). At the same time, they have imposed massive economic challenges and severely limited individual freedoms with possible large-scale consequences for mental health and well-being (7). After the lifting of lockdowns, European countries, including Italy, have been successful in limiting the incidence of infections throughout the summer, but a second epidemic wave has swept them since the beginning of the fall (8).

We retrospectively analyze the dynamics of COVID-19 since the emergence of the epidemic in Italy until September 30, 2020 through an age-structured Susceptible-Infectious-Recovered (SIR) model of severe acute respiratory syndrome coronavirus 2 (SARS-CoV-2) transmission calibrated on daily hospital admissions with a COVID-19 diagnosis recorded over the considered period. The aim of this work is to assess the health impact of the lifting of lockdown in Italy, providing counterfactual scenarios about alternative timing of reopening decisions and additional reopening of different educational levels and society. The burden of COVID-19 in the different scenarios is evaluated in terms of hospital and intensive care unit (ICU) admissions and bed occupancy.

## Results

### Baseline Model.

**Fig. 1.**
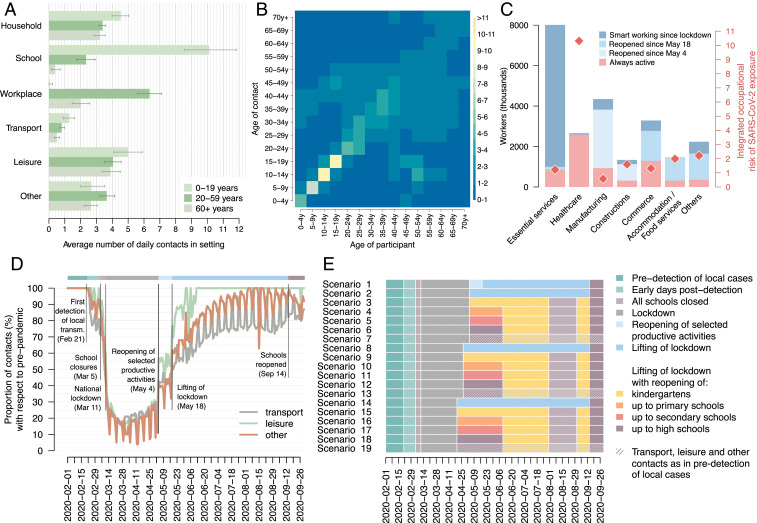
(*A*) Mean number (bars) and 95% CI (lines) of daily contacts by type of contact aggregated over three age groups (0 to 19, 20 to 59, and 60+ y old) as estimated from the analysis of the contact diaries collected in 2007 for the Italian population by the POLYMOD study (17). (*B*) Heat map of the overall contact matrix representing the mean daily number of contacts that an individual of a given age group has with other individuals, stratified by age group, used in the model to represent contact rates in the predetection epidemic phase. The color of each cell represents the mean total number of daily contacts (scale on the right). The contact matrix shown here is the mean of 300 bootstrapped contact matrices as obtained by the analysis of the contact diaries collected in 2007 for the Italian population by the POLYMOD study (17) (*SI Appendix*). (*C*) Workforce involved in different employment sectors who were physically present at work throughout the lockdown, worked from home since the lockdown, or were suspended and then reopened at different times (data from ref. 31); red diamonds represent the integrated occupational risk of exposure to SARS-CoV-2 in each sector (data from ref. 31; scale on the right *y* axis). (*D*) Proportion of contacts over time with respect to the preepidemic period in transportation means, leisure venues, and other generic settings, derived from refs. 32, 33 (*SI Appendix*). Main events and national government decisions for control of the COVID-19 epidemic are indicated. (*E*) Schematic representation of the timeline of different phases considered in the actual interventions (scenario 1) and in 18 counterfactual scenarios.

The model considers an age-specific susceptibility to infection (9) and is informed with detailed socioeconomic data to account for heterogeneity in contacts by age and settings (households; schools; workplaces; and in the community, further distinguished into transportation means, leisure venues, and other generic settings; Fig. 1 *A* and *B*), changes of work attendance before and during the lockdown (March 11) and in the progressive reopening phases (May 4 and 18; Fig. 1*C*), heterogeneity in the risk of infection in the different employment sectors (Fig. 1*C*), and changes of contacts in the community over time (Fig. 1*D*). To reproduce the introduction of infection precautions (face masks, hand hygiene, surface sanitation), the model includes stepwise changes in transmission rates during three disjoint periods: the early days of transmission (from the start of simulations to February 20); the early days of response and the lockdown (February 21 to May 3); and the reopening phase (May 4 to the end of simulations).

**Fig. 2.**
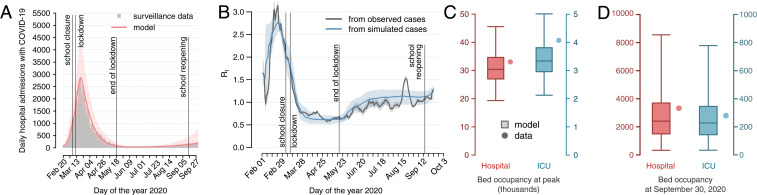
(*A*) Daily hospitalizations with COVID-19 over time in Italy, according to surveillance data (10) (gray bars) and as estimated by the baseline model, scenario 1 (solid line, median; shaded area, 95% CI). (*B*) Comparison of estimates of the net reproduction number R_t_, averaged over a weekly moving window, obtained from the daily number of symptomatic cases by date of symptom onset from surveillance data (10) (black solid line, median; shaded areas, 95% CI) and from estimates of the baseline model, scenario 1 (blue solid line, median; shaded areas, 95% CI). (*C*) Peak hospital and ICU bed occupancy by patients with COVID-19 according to official data (11) (dots) and corresponding baseline model estimates (boxplots: median, interquartile ranges, and 95% CI). (*D*) Hospital and ICU bed occupancy by patients with COVID-19 on September 30, according to official data (11) (dots) and corresponding baseline model estimates (boxplots: median, interquartile ranges, and 95% CI).

The simulated epidemic matches well with the national curve of daily hospital admissions with confirmed SARS-CoV-2 infection (10) over the whole study period (Fig. 2*A*). In addition, the model is validated by comparing model estimates against data and epidemiological quantities that have not been used for model calibration. Specifically, the resulting temporal profile of the net reproduction number R_t_ in simulated epidemic curves is strikingly close to the one directly estimated from the observed curve of cases by date of symptom onset, as reported to the national surveillance system (10) (Fig. 2*B*; see *SI Appendix* for details). Model estimates suggest that R_t_ dropped below the critical threshold of one in about 2 wk after the national lockdown on March 11, consistent with observations from data (6); afterward, R_t_ remained systematically below one during the lockdown period and then progressively increased to values close to one after the lifting of the lockdown on May 18; after the reopening of schools on September 14, R_t_ started to increase again. The model additionally reproduces well the number of hospital and ICU beds occupied at the peak (Fig. 2*C*) and at the end of simulations (Fig. 2*D*) (11). The model estimates an overall attack rate in the Italian population of 4.78% (95% CI: 2.01 to 10.51%) on September 30, 2020 and an average ascertained proportion of infections equal to 9.4% (95% CI: 4.3 to 22.4%) until June 30 [similarly to previous estimates for Italy (12)], and of 24.5% (10.5 to 58.0%) between July 1 and September 30. Furthermore, the estimated age-specific profile of the attack rate is consistent with that observed in a large-scale seroprevalence study in Spain (13) and estimated in most European countries (14) (*SI Appendix*). We estimated that, soon after the identification of the first COVID-19 case in Italy (February 21), transmission rates decreased by 30% (95% CI: 14 to 43%), likely due to the awareness of the population and the scale-up of local interventions (e.g., mandatory use of masks and adoption of hand sanitizers for supermarket clients). From May 4 onward, we estimate a 44% (95% CI: 36 to 52%) reduction of transmission rates with respect to predetection levels, possibly ascribable to increased mask usage, sanitation precautions in reopened commercial activities (bars, shops), and increased impact of contact tracing operations, possibly determined by lower incidence of cases. These estimates on reductions of the transmission rates are in agreement with previous independent estimates on the Italian context (12, 15).

### Impact of Interventions.

**Fig. 3.**
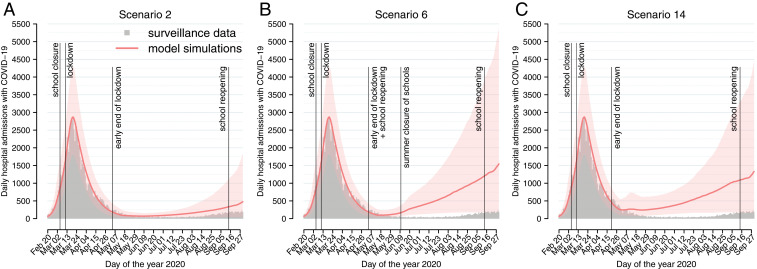
Daily hospitalizations with COVID-19 over time in Italy, according to surveillance data (10) (gray bars) and as estimated in (*A*) scenario 2 (end of lockdown anticipated to May 4), (*B*) scenario 6 (end of lockdown anticipated to May 4 + reopening of all educational levels), and (*C*) scenario 14 (end of lockdown anticipated to April 20). Solid line, median; shaded area, 95% CI.

Model estimates calibrated on the actually implemented interventions (scenario 1) were compared against 18 counterfactual scenarios aimed at showing what would have occurred under different circumstances (Fig. 1*E* and Table 1). We found that anticipating the lifting of the lockdown on May 4 (scenario 2; Table 1 and Fig. 3*A*) would have resulted in 25,997 (95% CI 8,189 to 66,114) cumulative hospitalizations between May 4 and September 30, corresponding to 14,078 (bootstrap 95% CI: 13,866 to 14,382) excess hospitalizations compared to the actual interventions, that is, a +118% increase in cumulative incidence. We would additionally expect 1,498 (bootstrap 95% CI: 1,468 to 1,524) excess ICU admissions with respect to the actual interventions. This additional burden is due to the earlier stabilization of the net reproduction number close to one, at a time characterized by higher incidence levels.

**Table 1.** Characteristics of considered scenarios and simulation results

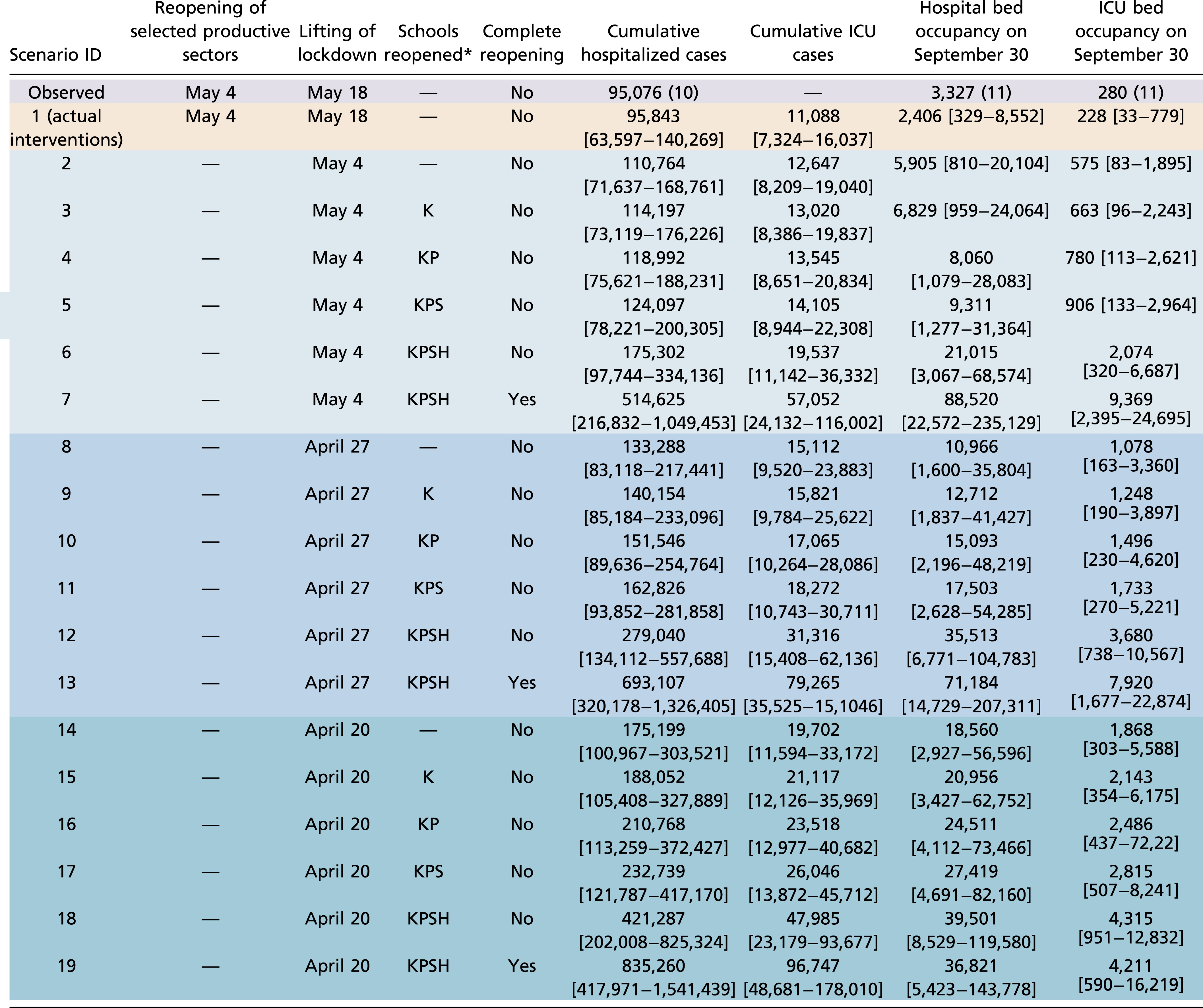

For all scenarios, all educational levels are closed on March 5 and reopened on September 14, and national lockdown initiates on March 11. For scenarios reopening schools after lockdown, the natural summer break is assumed between June 10 and September 14 for all educational levels except kindergartens, which are assumed to be closed between August 1 and September 14. Median and 95% CI are reported for the cumulative number of patients admitted to a hospital or ICU before September 30, and the hospital and ICU bed occupancy on September 30. Purple represents observed values, pink is the scenario representing actual interventions, and different shades of blue differ by the date of lifting of lockdown.

* K, kindergartens; P, primary; S, secondary; H, high schools. Reopening is assumed on the same day the lockdown is lifted.

The additional reopening of educational levels up to secondary schools from May 4 to September 30 (scenarios 3 to 5) would have had a limited impact on overall hospitalizations and ICUs (Table 1); however, including the reopening of high schools (scenario 6; Table 1 and Fig. 3*B*) would result in an excess of 77,401 (bootstrap 95% CI: 76,362 to 78,816) hospitalizations (+649% period increase) and 8,317 (bootstrap 95% CI: 8,173 to 8,411) excess ICU admissions compared to the actual interventions. The median ICU occupancy on September 30 in this scenario is estimated at almost 2,100 beds (over 10 times the one recorded at the same date), with a worst case of up to 6,700. For comparison, the current maximum national capacity is 8,800 ICU beds, of which 3,625 are COVID-19 dedicated.

Excess hospitalizations increased significantly with earlier reopening: Lifting the lockdown on April 27 while keeping schools closed (scenario 8; Table 1) would result in 36,703 (bootstrap 95% CI: 36,146 to 37,249) additional hospitalizations compared to the actual scenario, compared to the 13,648 estimated between April 27 and September 30 (+269% period increase); anticipating the end of lockdown at April 20 (scenario 14; Fig. 3*C*) would produce 78,375 (bootstrap 95% CI: 77,753 to 79,462) excess hospitalizations, compared to the 16,604 estimated between April 20 and September 30 (+472%). Combining the ending of lockdown on April 20 with reopening of all schools (scenario 18; Table 1) would have further amplified the additional health burden of COVID-19: In this case, we estimate 325,352 (bootstrap 95% CI: 323,702 to 329,399) excess hospitalizations, that is, an over 20-fold period increase, and 36,966 (bootstrap 95% CI: 36,619 to 37,401) excess ICU admissions. In the scenarios of a complete reversal to prepandemic contacts (scenarios 7, 13 and 19; Table 1), a massive second wave would have been experienced right after reopening. The temporal dynamics of counterfactual scenarios not shown in Fig. 3 are reported in *SI Appendix*.

### Fall Projections.

**Fig. 4.**
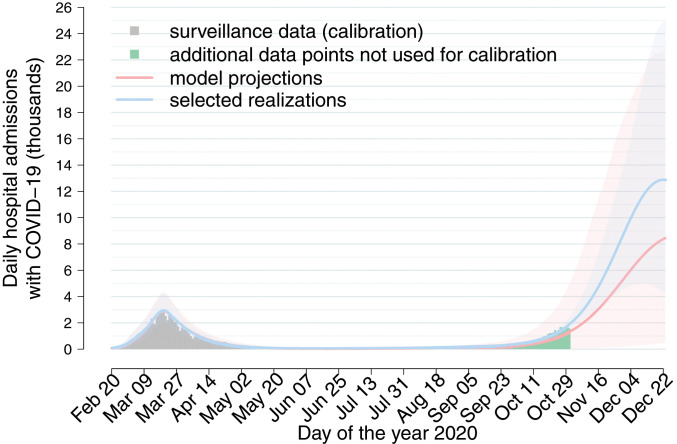
Daily hospitalizations with COVID-19 (thousands) over time in Italy, according to surveillance data (10) (gray bars, used for calibration; green bars, additional data points) and as projected under the assumption that the reopening of all educational levels and community contacts are maintained unchanged until December 23, without further control interventions. Red indicates projections from 10,000 model realizations; blue indicates the subset of 1,000 simulations with highest Poisson likelihood over hospital admissions occurring between September 15 and October 31. Solid line, median; shaded area, 95% CI.

We projected the number of hospitalizations under the actually implemented interventions expected until December 23, including the reopening of schools on September 14 and assuming no further interventions. Although the net reproduction number was already above the epidemic threshold by the beginning of September (Fig. 2*B*), school reopening in a context when almost all community contacts have been resumed (Fig. 1*D*) and with a relatively higher incidence in the community may hasten the epidemic growth and result in a large second wave (Fig. 4). Hospitalization data for the month of October were not used during calibration; thus the confidence intervals of these projections are wide. By restricting projections to those best reproducing hospital admissions between September 15 and October 31, we project a peak of ∼13,000 (range 5,000 to 25,000) hospitalizations per day in absence of further interventions, that is, a more than four-fold incidence compared to the peak observed during the first wave (Fig. 4).

### Subnational Analysis.

In Italy, there was a clear north−south gradient in the burden of SARS-CoV-2 infection during the first wave, which, however, was not associated with a different pathogen transmissibility (6, 16) but rather with different timings of introduction of the virus and relative timing of interventions. To evaluate possible effects of subnational heterogeneities on the effect of reopening strategies, we calibrated the model to subnational data, taking Lombardy, Lazio, and Campania as representative regions for northern, central, and southern Italy. These three regions represent over one-third of the Italian population and are also home to the three largest metropolitan areas in Italy (Milan, Rome, and Naples, respectively). The model was equally able to reproduce the observed epidemiological trend of daily hospital admissions in each region (10), with estimated posterior distributions of parameters at the regional level being largely compatible with national ones (*SI Appendix*).

**Fig. 5.**
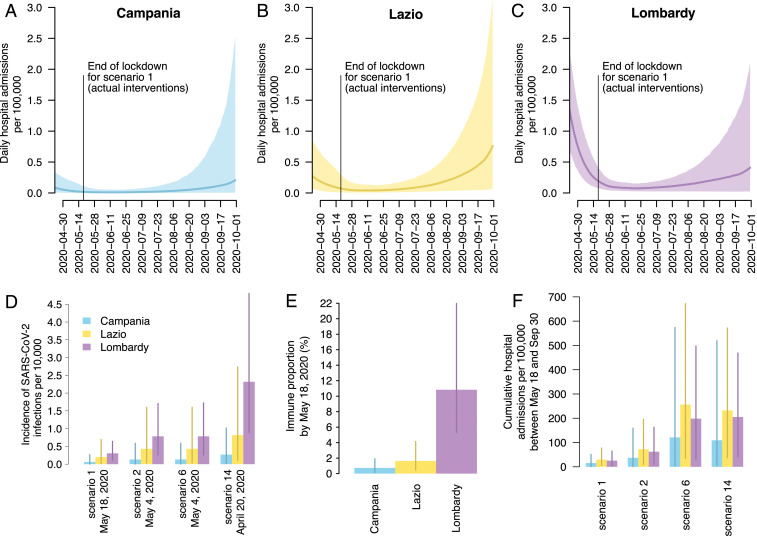
Subnational analysis for Campania, Lazio, and Lombardy. (*A*–*C*). Model estimated daily hospital admissions per 100,000 individuals under scenario 1 (actual interventions) after the lifting of lockdown in the three regions (solid lines, median; shaded area, 95% CI). (*D*) Model estimated incidence of infection per 10,000 individuals at the date of lifting of lockdown for selected scenarios (mean and 95% CI). (*E*) Estimated proportion of immune individuals on May 18 (mean and 95% CI). (*F*) Model estimated cumulative hospital admissions per 100,000 individuals under selected scenarios (mean and 95% CI).

The effect of reopening is projected to be heterogeneous across regions in terms of cumulative hospitalization rates per 100,000 population (Fig. 5 *A*–*C*). In particular, the infection incidence and the immune fraction at the end of lockdown are estimated to be highest in Lombardy and lowest in Campania (Fig. 5 *D* and *E*). This results in a projection of cumulative hospital burden until the end of September that is expected to be lowest in Campania (Fig. 5*F*), due to a combination of low infection incidence at reopening (Fig. 5*D*) and a comparatively younger demographic (*SI Appendix*). Lombardy, on the other hand, would likely have the benefit of some reduction of transmissibility due to a nonnegligible fraction of immune individuals estimated by the model (about 11%, compared to 2% in Lazio and 1% in Campania, Fig. 5*E*), so that its cumulated hospitalization incidence is lower than Lazio (Fig. 5*F*), despite much higher incidence levels at reopening.

## Discussion

Our analysis shows that the governmental decision to reactivate selected production sectors between May 4 and May 18 before lifting most of the lockdown restrictions maintained the reproduction number at low levels (around 0.50 to 0.70), allowing the prevalence of infection to decrease to lower levels. After May 18, the reproduction number rose progressively to a value slightly above one until mid-September, thereby resulting in a slowly growing incidence. According to our simulations, anticipating the lifting of lockdown on May 4 would have resulted in an approximately twofold incidence (+118%) of hospital admissions over the period May 4 to September 30. This effect was even more marked if lockdown restrictions had been lifted on April 20, with an almost sixfold incidence of hospitalization (+472%) over more than 5 mo. This highlights the importance of timing in exit strategies from lockdown during COVID-19 epidemics, suggesting that a successful reopening requires two critical conditions: a low value of the reproduction number and a sufficiently low incidence of infection. The first is needed to allow some margin for R_t_ to expand after the lifting of restrictions; the second is needed because the level of incidence will be approximately maintained constant after the reproduction number has grown to values close to one. This interpretation is confirmed when analyzing epidemiological trends across regions in the postreopening phase; regional differences in hospitalization rates are associated with the epidemiological conditions after the lifting of lockdown (e.g., prevalence of infection and prevalence of immunity in the population), without the need for assuming changes in the underlying structure of contacts or transmission parameters.

We suggest that, under low prevalence levels and the reduced overall transmission rates estimated for Italy in the postlockdown phase, the reopening of lower educational levels (up to secondary schools) in spring might have had a marginal effect on the burden and reproduction numbers of SARS-CoV-2. However, the effect of school reopening may have been larger when SARS-CoV-2 incidence in the community was more sustained, and thus individual-level strategies (e.g., case isolation, contact tracing) may be less effective. Potential challenges for public health ensuing from transmission in schools are also highlighted by our finding that reopening of all educational levels in spring (including high schools) might have had a major impact on the expected burden despite the limited time frame over which schools would reopen (5 wk to 7 wk, depending on the considered scenario). This result is also reflected in our projections of a brisk acceleration of transmission during the fall due to school reopening under an almost complete resuming of social activity (Fig. 1*D*) and given increased levels of community incidence. However, we note that we are unable to quantify the effect of protocols adopted to reduce transmission within school settings, such as reactive quarantine of classes, mask usage, physical distancing among students, promotion of hand hygiene, air ventilation of rooms, and improved sanitation of surfaces. In addition, classmates often have frequent contacts among themselves outside school buildings, for example, on transportation means, during study groups, or in non−school-related activities such as team sports. These contacts are not recorded as additional contacts in the POLYMOD study (17), since only the primary setting associated with a given contact is recorded. Therefore, the adopted contact matrix cannot distinguish transmission occurring properly within educational buildings from that related to activities associated with school reopening. We do not explicitly consider a potential increase in community contacts occurring among unemployed and suspended workers or students not attending schools. Finally, our projections do not take into account interventions being taken by the national and local governments to contrast the ongoing second wave (8).

Our findings highlight the importance of maintaining smart working for all job types for which it is sustainable: In a scenario where we assume that, on May 4, schools were reopened and all workers got physically back to work (thereby resuming the amount of social contacts at prepandemic levels), even in presence of a significant reduction of the transmission rates after release of lockdown, the COVID-19 disease burden was expected to escalate quickly, with a median of 57,000 (and up to 116,000) cumulative ICU admissions by September 30 (Table 1).

The observed outcome of the Italian postlockdown exit strategy depended on the reduction of overall transmissibility allowed by the adaptation of human behavior (e.g., adoption of personal protective equipment, improved hand hygiene, social distancing, and adherence to governmental indications), societal organization (e.g., reducing human density and improving sanitation in shops, restaurants, and public transport; specific adjusted risk management at workplaces; and increase of smart working) and public health prevention measures (e.g., tracing, testing, and isolation of contacts of cases; improvement of infection control procedures in hospitals and long-term care facilities; and systematic testing of health care workers independently of the presence of symptoms). Although these factors likely improved continuously over time, they were summarized in the model as stepwise changes in the transmission rates, due to the lack of more granular data and to avoid overfitting issues in parameter calibration. Despite this approximation, the model was able to capture with excellent accuracy the observed temporal changes of the effective reproduction number. In particular, there were insufficient data to explicitly model the isolation of positive individuals (in hospitals or at home) and the precautionary quarantine of case contacts following tracing activities. We acknowledge the limitation that the effectiveness of these interventions in reducing transmission may be dependent on the prevalence of infection. We estimate that the case ascertainment ratio during the summer, a period of low incidence, was about 25%, compared to less than 10% until June 30. The higher case ascertainment allowed by a limited circulation of the virus likely contributed to interrupt transmission chains and to maintain the net reproduction number close to the epidemic threshold. In counterfactual scenarios with higher incidence, we might expect the contribution of contact tracing to the reduction of transmissibility to be limited by the saturation of available resources; thus, our estimate of the excess hospitalizations and ICU admissions may be optimistic.

During the lockdown, a number of work activities in each considered employment sector remained open, as they were necessary for the maintenance of Italy’s basic needs. The successive reopening, between May 4 and May 18, of work activities in selected critical sectors for the country’s economy (construction sites and manufacturing industries) did not result in a significant additional burden of SARS-CoV-2 transmission. We acknowledge limitations in the quantification of the integrated occupational risks in some work sectors that may be characterized by specific prevention and protection measures (e.g., the use of personal protective equipment and infection control precautions among health care workers), or by specific work environment conditions (such as low temperature, high humidity, and great aerosolization in meat processing plants). However, sensitivity analyses with respect to modifications in the integrated occupational risks show that our conclusions are robust with respect to these limitations (*SI Appendix*). We note that the definition of the integrated occupational risks is strictly related to country-specific production systems and processes, organizational and hygiene regulations, and practices related to housing and transportation of workers; therefore, the specific integrated occupational risks defined in this work cannot be directly generalized to other countries.

Additional model limitations are linked to the persisting uncertainties on the contribution of children to overall transmission. There seems to be a consensus that children are less susceptible to infection given exposure [between 29% and 69% compared to adults, according to a review of eight different contact tracing studies (18)], and recent analyses found no difference in infectiousness between children and adults (19, 20). Several studies found equivalent viral loads in children and adults (21–24), and transmission in schools (25) and other youth settings (26) has been now widely documented. The assumptions of our baseline model are consistent with these findings; however, our conclusions remained qualitatively robust when considering alternative hypotheses on child infectiousness and susceptibility to infection, and relevant deviations occurred only for scenarios including the opening of high schools (*SI Appendix*). We did not consider possible differences between the infectiousness of asymptomatic and symptomatic cases. The viral load in symptomatic and asymptomatic individuals seems to be similar (27–29); while symptomatic individuals may be shedding more virus per unit time due to coughing and other respiratory symptoms, on the other hand, they may expose a smaller number of contacts, due to the higher probability of being detected and isolated (whether at home or in a hospital). We stress the need for updated contact surveys in Italy, since the only available direct data on age-specific mixing patterns were collected in 2007 (17). Finally, we did not take into account possible changes over time in hospital admission probabilities. In principle, there might have been changes in criteria related to the severity of symptoms requiring hospital admission in periods and regions of severe hospital strain. However, we expect that such local and temporally limited changes had a minor effect on hospitalization rates at the national level; this is reflected in the model’s ability to correctly reproduce at the same time both trends in hospitalizations (Fig. 2*A*) and trends in transmissibility (Fig. 2*B*) with a temporally constant hospitalization rate.

This study provides a framework for assessing the health impact of exit strategies from the COVID-19 lockdown, based on data-driven modeling of social contacts, work attendance, integrated occupational risks, human mobility, and time use. Similar approaches, with different focus on the types of modeled contacts, have been previously proposed to model COVID-19 exit strategies (30). The proposed model structure does not realistically reproduce contacts in individual households, schools, and workplaces, and therefore does not allow for specific inference on the contribution to transmission of each of these routes. It should rather be interpreted as a robust framework to model the temporal evolution of epidemiological trends at the national level via changes in age-specific contact patterns. The insights provided here reinforce the need to wait for interventions to bring the infection prevalence to low levels before reopening productive and societal sectors, and caution against complete resuming of prepandemic social dynamics (including physical attendance at work for jobs that can be executed remotely) even in the presence of important reductions in the transmission rates.

## Materials and Methods

### Baseline Model.

We use a mathematical model of SARS-CoV-2 transmission, informed with detailed socioeconomic data on 1) age-structured contact rates, which allow estimating contacts relevant to the transmission of SARS-COV-2 in the most critical settings; 2) human mobility and time use, which allow estimating how contacts into transportation means (stations, trains, buses, taxis, etc.), leisure venues (restaurants, bars, discos, sport facilities, concerts venues, museums, parks, etc.), and other generic settings (shops, offices, banks, etc.) have changed over time; 3) work attendance over time (as determined by implemented mitigation policies, e.g., suspension of certain employment sectors, smart working) and integrated occupational risk by employment sector, which allows characterization of the risk of infection in the different employment sectors (health care, manufacturing, etc.). Contacts within households were assumed to not change over time, while school contacts are regulated by implemented policies on school closure.

The transmission model is an age-structured SIR model with a gamma-distributed generation time with mean 6.6 d (27) and operates at the country level (however, a regional implementation of the model was also simulated to evaluate interregional epidemiological heterogeneities). The model includes contacts in multiple settings, such as households, schools, workplaces, and in the community (further distinguished into transportation means, leisure venues, and other generic settings; Fig. 1 *A* and *B*) (17). Workers are disaggregated into seven employment sectors (essential services, health care, manufacturing, commerce, constructions, accommodation/food services, and others) and are assumed to have contacts at work based on official data on age-specific workplace attendance in the different sectors before and after lockdown. For each sector, we considered an associated occupational risk of SARS-CoV-2 exposure, estimated by the Italian Workers’ Compensation Authority (Fig. 1*C* and *SI Appendix*) (31). Occupational risks by employment sector were obtained by combining three different indexes, estimated in ref. 31: 1) the exposure index, that is, the likelihood to be in contact with potential sources of infection during work activity; 2) the proximity index, that is, the intrinsic features of work activity that cannot guarantee an adequate social distancing; 3) the aggregation index, that is, work activities conditions that determine contacts with people other than workmates.

We used publicly available data on human mobility (32) and time use (33) to modulate temporal changes in community contacts ensuing from both spontaneous behavioral response to risk perception by individuals and governmental interventions (Fig. 1*D* and *SI Appendix*). Fig. 1*D* also shows key events of the COVID-19 epidemic in Italy, from the detection of the first COVID-19 case in Italy (February 21) to the national closure of schools (March 5), the national lockdown (March 11), the gradual lifting of the lockdown (May 4 and 18), and the reopening of schools in the fall (September 14).

We considered an age-specific susceptibility to SARS-CoV-2 infection (i.e., the probability of developing infection upon effective exposure to an infectious case with respect to a reference age group). Specifically, we used the posterior distributions estimated by Zhang et al. (9): taking the age group 15 y to 64 y as the reference, we consider an average relative susceptibility of 0.33 (95% CI: 0.24 to 0.47) for children under 15 y of age, and 1.47 (95% CI: 1.16 to 2.06) for older adults (above 65 y). These values are in line with those reported in seven other independent studies reviewed in ref. 18. We assume the same infectiousness across individuals of different ages.

A scaling factor for transmission in the days preceding the detection of the first COVID-19 case in Italy (February 20) was computed using the next-generation matrix approach (34) in such a way as to match the reproduction number at the onset of the COVID-19 epidemics in Italy, estimated at about three (6, 16, 27), in line with estimates from other parts of the world (30, 35–38). The reductions in transmission rates in the two successive periods (until the end of lockdown and after reopening) were free model parameters estimated via calibration.

The transmission model provides estimates of the age-specific daily incidence of SARS-COV-2 infections from February 1, 2020 (20 d before the first case of local transmission was confirmed by the Italian authorities) up to September 30, 2020. We used information on 1) age-specific probability of developing respiratory symptoms (39), 2) probability of requiring intensive care (40), 3) delays between symptom onset and hospitalization and between hospitalization and admission to ICU (40), and 4) length of stay in hospital and ICU (40) to estimate the daily incidence of cases admitted to the hospitals and ICU and the daily number of occupied hospital and ICU beds. The probability of hospitalization for individuals with respiratory symptoms was a free model parameter estimated via calibration.

The baseline model accounting for the governmental interventions (scenario 1; Fig. 1*E*) was calibrated by using a Markov chain Monte Carlo approach applied to the Poisson likelihood of observing the actual daily number of COVID-19 hospital admissions until September 30 (10), as recorded by the national surveillance system (described in ref. 16).

Modeling details are reported in *SI Appendix*.

### Impact of Interventions.

To evaluate the impact of governmental interventions (scenario 1), we compared epidemiological outcomes experienced under actual interventions with those obtained under a number of counterfactual scenarios where the dates of reopening decisions are anticipated and considering the additional reopening of different educational levels and society (Fig. 1*E*). In particular, in scenario 2, we simulate the epidemic trajectory by assuming that the lifting of lockdown is anticipated from May 18 to May 4, skipping the intermediate phase of reopening selected productive activities; in scenarios 3 to 6, we additionally consider the progressive reopening of educational levels (from kindergarten to high schools); in scenario 7, we assumed that all leisure, transport, and other community contacts, as well as work attendance and school openings, revert to the prepandemic situation instantaneously after the end of lockdown (“complete reopening”); in scenarios 3 to 7, schools close for the summer break on June 10 and kindergartens close on July 30; scenarios 8 to 13 and 14 to 19 replicate the same assumptions as scenarios 2 to 7, but further anticipate the date of lifting of lockdown by 1 and 2 wk, respectively (on April 27 and 20); all educational levels reopen in all scenarios on September 14.

### Fall Projections.

We project model simulations until December 23 to simulate the impact of school reopening in absence of further interventions, using 10,000 model realizations. Because of the wide variability of the CIs, we additionally project the subset of 1,000 simulations with the best Poisson likelihood of observing the recorded daily numbers of COVID-19 hospital admissions between September 15 and October 31; data from the month of October have not been used during calibration.

### Subnational Analysis.

We recalibrated the model to hospital admission data (10) in three large Italian regions (Campania, Lazio, and Lombardy), by adjusting inputs for the population age structure and the estimated basic reproduction number. Full details are reported in *SI Appendix*.

## Supplementary Material

Supplementary File

## Data Availability

Epidemic curves by date of symptom onset and hospital admission have been deposited in Zenodo (10.5281/zenodo.4300101).
