# Supplementary material for: Retrospective analysis of the Italian exit strategy from COVID-19 lockdown
Source: Proc Natl Acad Sci U S A. 2021 Jan 7;118(4):e2019617118. doi: 10.1073/pnas.2019617118 (PMC7848712; doi:10.1073/pnas.2019617118)
Supplement: Supplementary File [file pnas.2019617118.sapp.pdf]

SUPPLEMENTARY MATERIAL

Retrospective analysis of the Italian exit strategy from COVID-19 lockdown

Valentina Marziano [a,^], Giorgio Guzzetta [a,^], Bruna Maria Rondinone [b], Fabio Boccuni [b], Flavia Riccardo [c], Antonino Bella [c], Piero Poletti [a], Filippo Trentini [a], Patrizio Pezzotti [c], Silvio Brusaferro [c], Giovanni Rezza [c], Sergio Iavicoli [b], Marco Ajelli [d,e], Stefano Merler [a,*]

[a] Center for Health Emergencies, Bruno Kessler Foundation, Trento 38123, Italy;
[b] Department of Occupational and Environmental Medicine, Epidemiology and Hygiene, Italian Workers' Compensation Authority, Monteporzio Catone (Rome) 00078, Italy;
[c] Department of Infectious Diseases, Istituto Superiore di Sanità, Rome 00161, Italy;
[d] Department of Epidemiology and Biostatistics, Indiana University School of Public Health, Bloomington, IN 47405
[e] Laboratory for the Modeling of Biological and Socio-technical Systems, Northeastern University, Boston, MA 02115

[^] joint first authors
[*] corresponding author

Table of Contents

Overview of the baseline model for SARS-CoV-2 transmission **2**
Baseline model equations **2**
Computation of contact matrices from individual contact diaries **4**
Computation of the proportion of social contacts over time **5**
Sector-dependent integrated occupational risk of exposure to SARS-CoV-2 .... **6**
Model initialization **9**
Model output **9**
Estimate of the stage-specific relative infectiousness **10**
Estimate of the scaling factor for transmissibility **11**
Model calibration **12**
Additional results on the baseline model **13**
Sensitivity analyses **14**
Subnational analyses **20**
Computation of the net reproduction number **23**

Overview of the baseline model for SARS-CoV-2 transmission

We developed a transmission dynamics model of SARS-CoV-2 transmission, based on an age-structured stochastic susceptible-infectious-removed (SIR) scheme. The model includes contacts in multiple settings, such as home, schools, workplaces and in the community (further distinguished into transportation means, leisure venues and other generic settings). Contacts are informed by data for Italy made available by the POLYMOD study, a large-scale European contact survey [1]. The evolution over time of community contacts as a consequence of individual behavioral change and governmental interventions was assumed to be modulated by human mobility data made available by Google [2] and projected to the POLYMOD categories of transport, leisure and other generic settings using data from time use [3].

Workers are disaggregated into 7 employment sectors:

1. Essential Services (agriculture; energy; water and waste management; goods transportation and storage; information and communication; credit and insurance; professional and technical activities; public administration; education; caregivers and domestic activities);
2. Health Care (health care workers and family assistants for elderly);
3. Manufacturing (manufacturing; metallurgical and mining industry);
4. Commerce;
5. Constructions;
6. Accommodation/Food services
7. Others (e.g. real-estate agencies; rental and support services; cultural, sport and recreational enterprises).

In addition, we consider an eighth group of individuals who do not attend a workplace and therefore do not experience contacts with colleagues or customers; this includes unemployed, not gainfully occupied (including children), retired individuals, as well as workers in smart working mode or suspended by lockdown restrictions. Age-specific data on active workers in the different sectors before and after lockdown [4], including information on smart work prevalence [5], were estimated by the Italian Workers' Compensation Authority (INAIL) integrated with the results of Italian National Survey on Occupational Safety and Health (INSuLa) [6, 7].

For the purpose of disease transmission, smart working was considered equivalent to work suspension and unemployment, as it does not entail social interactions with colleagues or customers.

The model considers three consecutive infectious compartments with different levels of infectiousness [8], in order to reproduce a gamma-distributed generation time with average 6.6 days [9, 10]. We considered 20 age groups (19 5-year age groups from 0 to 94 years and one age group for individuals aged 95 years or older). Children (0-14 years old) were considered less susceptible to infection upon exposure compared to the bulk of the adult population (aged 15-64 years), while the elderly were considered more susceptible [11]; in a sensitivity analysis, homogeneous susceptibility to SARS-CoV-2 infection across ages was considered. Workers were subject to a sector-dependent integrated occupational risk of SARS-CoV-2 exposure, taking into account heterogeneous exposure risks and interactions required by different jobs. In a sensitivity analysis, we considered homogeneous occupational risks across employment sectors. We assumed asymptomatic and symptomatic individuals to be equally infectious, as suggested by an early analysis of virological data from Lombardy [9] and Veneto [12]. Individuals of different ages were also assumed to be equally infectious; in a sensitivity analysis, we considered that children may be half as infectious as adults. Finally, we assumed that recovered individuals are immune to re-infection, considering the short time frame of simulations (February 1 to December 23, 2020) and the relatively low attack rate of SARS-CoV-2 (and therefore of population immunity) after the first COVID-19 epidemic wave documented in hardly hit countries [13, 14].

Baseline model equations

The population is divided in 160 classes (20 age groups x 8 employment types, including the 7 employment sectors reported above plus the group of unemployed, not gainfully occupied, retired, smart-workers and suspended workers). Infectious contacts within and between classes may occur in 6 different settings (home H, schools S, workplaces W, transportation means T, leisure venues L and other generic places O), and are combined in an overall contact matrix according to the following equation:

$$\text{[Eq1]} \quad M^{e}_{a,\tilde{a}}(t) = H_{a,\tilde{a}} + \alpha^{S}_{a}(t)S_{a,\tilde{a}} + \alpha^{T}_{a}(t)\alpha^{T}_{\tilde{a}}(t)T_{a,\tilde{a}} + \alpha^{L}_{a}(t)\alpha^{L}_{\tilde{a}}(t)L_{a,\tilde{a}} + \alpha^{O}_{a}(t)\alpha^{O}_{\tilde{a}}(t)O_{a,\tilde{a}} + E^{e}_{a,\tilde{a}}$$

where:

- $M^e_{a,\tilde{a}}(t)$ represents the age-group and employment-specific contact matrix, whose entries describe the mean numbers of persons in age group $\tilde{a}$ encountered by an individual of age group a and employment group e per day;
- $\alpha^C_a(t)$ represents the fraction of individuals in age group a who experience contacts in setting $C \in \{S, T, L, O\}$; this value changes over time depending on individual behavior changes and governmental decisions on restrictions applied to the community (e.g. national lockdown) and closure/reopening of schools (see below);
- $H_{a,\tilde{a}}, S_{a,\tilde{a}}, T_{a,\tilde{a}}, L_{a,\tilde{a}}, O_{a,\tilde{a}}$ are the mean number of individuals of age $\tilde{a}$ contacted per day by an individual of age a in the settings described above. These matrices are computed from individual contact diaries collected during the POLYMOD study [1] (see next section); school closures were modeled by simply removing school contacts for individuals attending the closed educational level;
- $E^e_{a,\tilde{a}}$ is defined as

$$\text{[Eq2]}\quad E^e_{a,\tilde{a}} = \begin{cases} 0 & if\ e = unemployed, not\ gainfully\ occupied, retired, smart\ working, suspended \\ k\ \alpha^{O}_{\tilde{a}}\ \rho_e \mathrm{W}_{a,\tilde{a}} & if\ e = commerce\ or\ accommodation/food\ services \\ k\ \rho_e \mathrm{W}_{a,\tilde{a}} & otherwise \end{cases}$$

where:

  - $W_{a,\tilde{a}}$ represents the average contacts per day at work that an individual of age a has with individuals of age $\tilde{a}$ as computed from individual POLYMOD diaries of working participants [1];
  - ρ_e is defined only for employed individuals and represents the integrated occupational risk estimated by INAIL in each professional sector e;
  - $\alpha^{O}_{\tilde{a}}$ reflects the impact of movement restrictions on the number of contacts with customers in workers from commerce and accommodation/food services sectors.
  - k is an adjusting factor used to guarantee the conservation of the overall number of contacts occurring at work as observed in the POLYMOD study [1], i.e. before the epidemics; in particular:

$$\text{[Eq3]}\quad k = \frac{\sum_a \sum_{\tilde{a}} \pi_a W_{a,\tilde{a}}}{\sum_a \sum_{\tilde{a}} \pi_a \sum_{\varepsilon} \xi_{\varepsilon,a}\ \rho_{\varepsilon}\ W_{a,\tilde{a}}}$$

  where:

    - π_a is the employment rate of age-group a (i.e. the proportion of employed individuals within age group a);
    - $\xi_{\varepsilon,a}$ is the proportion of workers that are employed in professional sector ε among those of age a.

  In this way, when the risk is assumed homogeneous across work sectors, i.e. ρ_{ε} = 1, we obtain k = 1; assuming pre-epidemics conditions ($\alpha^{O}_{\tilde{a}}$=1 in absence of restrictions), the matrix $E^e_{a,\tilde{a}}$ corresponds exactly to the POLYMOD contact matrix, as it should.

The force of infection for subjects of age a and employment type e is then modeled as follows:

$$\text{[Eq4]}\quad \lambda_{a,e}(t) = \beta(1-\varphi(t))r_a \sum_{\tilde{a}} M^e_{a,\tilde{a}}(t) \frac{\sum_{\tilde{e}} \chi_I I_{\tilde{a},\tilde{e}}(t) + \chi_J J_{\tilde{a},\tilde{e}}(t) + \chi_K K_{\tilde{a},\tilde{e}}(t)}{N_{\tilde{a}}}$$

where:

- β is a scaling factor shaping the transmissibility during the period before detection of the first local cases in Italy, on February 21;
- $\varphi(t)$ is a coefficient representing the reduction in transmissibility due to the effect of infection precautions taken spontaneously by the population, as well as due to public health regulations such as mandatory sanitation and mask use in public transport, supermarkets, bars and restaurants; it affects all kinds of contacts, including household contacts. We model this parameter with a piecewise constant function, as follows:

$$\varphi(t) = \begin{cases} 0 & \text{from the start of simulations to Feb 21} \\ \varphi_1 & \text{from Feb 21 to the end of lockdown (depends on scenario)} \\ \varphi_2 & \text{from the end of lockdown to the end of simulations} \end{cases}$$

φ_1 and φ_2 were free parameters estimated during calibration;

- r_a is the relative susceptibility to SARS-CoV-2 infection at age a, representing the relative probability of becoming infected given exposure; we used for the values of r_a the posterior distribution of values estimated in [11], with average 0.33 (95%CI 0.24-0.47) when $a < 15$; and 1.47 (95%CI 1.16-2.06) when $a \geq 65$ [11]; r_a was set at 1 (reference value) for intermediate ages.
- χ_I, χ_J, χ_K are the stage-specific relative levels of infectiousness;
- $N_{\tilde{a}}$ represents the total number of individuals in age group $\tilde{a}$.

Transitions across different epidemiological classes can be summarized by the following differential systems:

$$\text{[Eq5]} \quad \begin{cases} S'_{a,e}(t) = -\lambda_{a,e}(t)\, S_{a,e}(t) \\ I'_{a,e}(t) = \lambda_{a,e}(t) S_{a,e}(t) - \gamma I_{a,e}(t) \\ J'_{a,e}(t) = \gamma I_{a,e}(t) - \gamma J_{a,e}(t) \\ K'_{a,e}(t) = \gamma J_{a,e}(t) - \gamma K_{a,e}(t) \\ R'_{a,e}(t) = \gamma K_{a,e}(t) \end{cases}$$

where:

- S represents the number of individuals susceptible to SARS-CoV-2 infection;
- I, J, K represent the number of individuals in the three stages of infection; in particular, I represents the initial stage of infection, while J and K reflects the peak and the declining phase of infectiousness;
- R represents the number of individuals who recover from the infection; we assumed that recovering from infection provides full immunity against re-infection for at least the duration of our simulations (less than one year);
- γ is the recovery rate associated with each stage of infection.

Simulation results discussed in the main text and in the following sections were obtained by using a stochastic version of the model described above.

Computation of contact matrices from individual contact diaries

We computed contact matrices from publicly available data on 831 individual diaries collected in Italy during the POLYMOD survey [15]. Each diary reports information on all the social contacts experienced by a given participant on a single day of the survey (average number of independent contacts per participants: 19.77 [1]). Recorded information include the age and employment status of the participant, the ages of the contacted persons, the duration, frequency and proximity (either physical or non-physical) of each social interaction, and the location where this interaction occurred (choosing between home, work, school, transportation means, leisure venues and a residual generic category indicated with "other"). Contact matrices were obtained by weighing both weekday and weekend data from the POLYMOD survey. We grouped the ages of participants and contacts in 14 5-year age classes plus an additional class including all individuals aged 70 years or older.
For a given participant of age class a, his reported contacts $c^C_{a,\tilde{a}}$ were aggregated by the age class $\tilde{a}$ of the contacted person and by the location $C \in \{H, S, T, L, O, W\}$ where the contact took place, regardless of their frequency, duration and proximity. If the exact age of the contact was unknown by the participant, this was provided as a range; in these cases, we used the midpoint of the range to assign an age class to the contacted person as done in the original POLYMOD study [1].
To take into account sample variability, we computed 300 bootstrapped contact matrices. At each bootstrap iteration, we sampled with replacement 831 diaries, choosing the age of the participant with probability proportional to the age distribution of the Italian population [16]. Then, we computed the average number of contacts that a single individual of age group a experiences with individuals of age $\tilde{a}$ in a given setting $C \in \{H, S, T, L, O\}$ from the following equation:

$$[Eq6]\quad C_{a,\tilde{a}} = \frac{\sum_{P_a} c^C_{a,\tilde{a}}}{P_a}$$

where P_a is the number of sampled participants of age group a. For workplaces (i.e. C=W), the average number of contacts $W_{a,\tilde{a}}$ was computed as:

$$[Eq7]\quad W_{a,\tilde{a}} = \begin{cases} \frac{\sum_{Z_a} c^W_{a,\tilde{a}}}{Z_a} & if\ Z_a > 0 \\ 0 & otherwise \end{cases}$$

where Z_a is the number of sampled participants of age group a who reported "working" as employment status. For the purpose of the model, contacts reported with individuals aged 70 years or more were then re-distributed over 6 further age groups (70-74, 75-79, 80-84, 85-89, 90-94, 95+), proportionally to the population of each age group [16]. We assumed that participants within each of these subgroups had the same average number of contacts in a given setting with individuals of other ages as the overall group of participants aged 70 and older.

Computation of the proportion of social contacts over time, $\alpha^C_a(t)$

To estimate the proportion of social contacts occurring in the community over time, we combined mobility data for Italy made available by Google expressly for the COVID-19 emergency [2] with Italian time use data [3] estimated before the pandemic. Google data represent the daily time spent by individuals in different types of places as a differential proportion with respect to a pre-pandemic baseline average [2]. Time use data provide the average time spent during a day in different locations or activities [3].

We associated each category of time use (τ) to a type of community contact considered in the POLYMOD study ($C\epsilon\{Transport, Leisure, Other\}$, and to a type of place considered by mobility data (ν), according to Table S1. We then computed the relative contribution of each time use category (η_τ) to the time spent for a given type of community contact as considered in the POLYMOD study. Eventually, we computed $\alpha^C_a(t)$ as the sum of the Google mobility data g_ν associated to the corresponding time use category τ, $g_\nu[\tau]$, weighted by η_τ, obtaining $\alpha^C_a(t) = \sum_{\tau\epsilon C} \eta_\tau g_\nu[\tau]$. As a result, $\alpha^T_a(t)$ was set equal to Google mobility data for transit stations, $\alpha^O_a(t)$ was set equal to Google mobility data for retail and recreation, and $\alpha^L_a(t)$ was given by the weighted sum of Google mobility data for parks (with weight 54.0%) and retail and recreation (with weight 46.0%) (see Table S1). In absence of age-specific data, we assumed the same values of $\alpha^C_a(t)$ for all ages. The resulting values over time are reported in Figure 1D in the main text.

Table S1. Correspondence between categories in social contact, time use and human mobility data.

Type of community contact in POLYMOD (C) [15]	Time use category [3] (τ)	Average daily time spent in activity [3]	Relative weight in community contact (η_τ)	Type of place in Google mobility data [2] (ν)
Transport	Urban public transport	6'	0.6667	Transit stations
Transport	Long-haul transport	3'	0.3333	Transit stations
**Transport**	**Total**	**9'**	**1.000**	
Leisure	Restaurants/bars	20'	0.2222	Retail and recreation
Leisure	Sports	9'	0.0952	Retail and recreation
Leisure	Cultural	3'	0.0317	Retail and recreation
Leisure	Recreational	3'	0.0317	Retail and recreation
Leisure	Other leisure	2'	0.0159	Retail and recreation
Leisure	Religious	6'	0.0635	Retail and recreation
Leisure	Outdoors	49'	0.5397	Parks
**Leisure**	**Total**	**91'**	**1.000**	
Other	Commerce	23'	0.8000	Retail and recreation
Other	Services	6'	0.2000	Retail and recreation
**Other**	**Total**	**29'**	**1.000**	

Sector-dependent integrated occupational risk of exposure to SARS-CoV-2

For each economic sector, a risk assessment was carried out regardless of the prevention and protection measures put in place. These measures, including PPE, are part of the risk mitigation actions. The integrated occupational risk index (ρ_e) considers the likelihood to be in contact with potential sources of infection during the work activity, the intrinsic features of work activity which cannot guarantee an adequate social distancing and the condition linked to work activities that may determine contacts with people other than workmates.
A method to estimate the risk of exposure to SARS-CoV-2 in the workplace has been developed taking into account the specific characteristics of production processes and the work organization contributions to the risk and heterogeneous exposure to close contacts with external subjects (public, clients, etc.) required by different jobs.
This methodology is based on the general approach to risk analysis in the field of occupational safety and health (OSH) [17]. In this case, such approach is not strictly intended to mitigate harm for single work activity; instead, it is aimed at identifying the general integrated occupational risk levels for the working population by sector, in line with the strategy of the decision makers for the lifting of the containment measures.
In this framework, the occupational risk of exposure to SARS-CoV-2 might be classified based on three variables:

- Exposure: the likelihood to be in touch with a potential source of infection during the work activity. To quantify this parameter, we used the perception of exposure indicator as defined by the O'Net survey [18] adapted to Italian context by comparison with the indicator of biological risk for viruses or bacteria exposure already defined in the framework of the Italian Survey of Occupational Safety and Health at Work (INSuLa) based on a representative sample of national working population [6, 7], according to the scale: from 1 = "low probability" to 5 = "high probability".
- Proximity: the intrinsic features of work activity which do not guarantee an adequate social distancing. To quantify this parameter, we used the perception indicator of physical proximity to other people during the work activities as defined by the O'Net survey based on Standard Occupational Classification (SOC) adapted to the Italian system and graded according to the scale: from 1 = "work carried out alone almost throughout the working time" to "5 = work carried out in close proximity with others for most of the working time" [18].
- Aggregation: the kind of work activity that may determine contacts with other people other than with company's workers (restaurants, retail, entertainment, hospitality, education, etc.) defined as a factor in the following classes: 1.00 = "limited presence of a third party" (e.g. manufacturing sector, industry, offices that are not opened to the public); 1.15 = "intrinsic presence of third parties controlled through the organization" (e.g. retail, personal services, offices that are opened to the public, cafes, restaurants); 1.30 = "aggregations controllable with procedures" (e.g., health care, schools, prisons, army, public transports); 1.50 = "large aggregations not easily controlled by specific procedures" (e.g. shows, sport events) [6].

Both exposure and proximity average values have been calculated for each employment sector according to the Italian Classification of Economic Activities (ATECO), the equivalent of European Classification of Economic Activities (NACE) [19]. Both exposure and proximity average values were normalized using the minimum and maximum value equation:

$$\text{[Eq8]} \quad y_i = \frac{x_i - x_{min}}{x_{max} - x_{min}}$$

where y_i is the standardized score, x_i is the original rating score, x_{min} is the lowest possible score on the rating scale used, and x_{max} is the highest possible score on the rating scale.
The aggregation factor has been defined for each employment sector. The final product defines the risk levels (RL) in the following four classes: Low RL < 2; Medium-Low 2 < RL < 4; Medium-High 4 < RL < 8; High RL > 8.
Furthermore, updated data on the workforce [4] were associated with each activity sector to obtain a burden of risk levels related to the number of potential exposed workers. We used commuting variables taken from the Italian National Survey on Occupational Safety and Health (INSuLa) [6, 7] (percentages of use of public transportation and average times of commuting) stratified by gender, age and geographical area to evaluate the impact on mobility due to the re-opening of most activities and to the workers commuting. The final integrated risk index is the product of the normalized exposure score, the normalized proximity score, and the aggregation factor (Table S2).

Such integrated risk classification appears coherent with the compensation claims application data available at National level for each employment sector [20].
Risk factors for the 7 professional sectors considered in the model were obtained by aggregating risks estimated for 20 professional subcategories (shown in Table S2). The values of ρ_e were obtained by computing the average from represented subcategories in each employment sector e, weighted by the number of active workers of each subcategory (shown in Table S3).

Table S2. Integrated occupational risk levels, standard deviations and risk classes by NACE employment sectors

Description of employment sectors		Risk level	SD	Risk class
A	Agriculture, Forestry And Fishing	1.00	1.19	Low
B	Mining And Quarrying	0.70	0.67	Low
C	Manufacturing	0.58	0.68	Low
D	Electricity, Gas, Steam And Air Conditioning Supply	0.57	0.54	Low
E	Water Supply; Sewerage, Waste Management And Remediation Activities	1.99	1.66	Low
F	Construction	1.31	1.24	Low
G	Wholesale And Retail Trade; Repair Of Motor Vehicles And Motorcycles	1.60	1.89	Low
H	Transportation And Storage	1.54	1.69	Low
I	Accommodation And Food Services Activities	1.99	1.24	Low
J	Information And Communication	0.55	0.59	Low
K	Financial And Insurance Activities	0.33	0.34	Low
L	Real Estate Activities	0.36	0.18	Low
M	Professional, Scientific And Technical Activities	0.91	1.36	Low
N	Administrative And Support Services Activities	1.49	1.13	Low
O	Public Administration And Defence; Compulsory Social Security	4.82	2.93	Medium-High
P	Education	2.67	1.75	Medium-Low
Q	Human Health And Social Work Activities	12.19	3.58	High
R	Arts, Entertainment And Recreation	2.08	1.61	Medium-Low
S	Other Services Activities	3.68	2.70	Medium-Low
T	Activities of Households as Employers; Undifferentiated Goods and Services Producing Activities of Households for Own Use	5.14	2.13	Medium-High

Table S3. Number of active workers before and after the lockdown and employment sectors for aggregation.

NACE EMPLOYMENT SECTORS	ACTIVE WORKERS		EMPLOYMENT SECTOR IN THE MODEL
	BEFORE LOCKDOWN	AFTER LOCKDOWN	
Agriculture, Forestry And Fishing	908,780	854,060	Essential services
Mining And Quarrying	24,740	9,730	Manufacturing
Manufacturing	4,321,420	1,444,130	Manufacturing
Electricity, Gas, Steam And Air Conditioning Supply	114,150	114,150	Essential services
Water Supply; Sewerage, Waste Management And Remediation Activities	242,780	242,780	Essential services
Constructions	1,339,370	523,690	Constructions
Wholesale And Retail Trade; Repair Of Motor Vehicles And Motorcycles	3,286,500	1,804,120	Commerce
Transportation And Storage	1,142,740	1,142,740	Essential services
Accommodation And Food Services Activities	1,480,190	317,590	Catering and lodging
Information And Communication	618,120	618,120	Essential services
Financial And Insurance Activities	635,590	635,590	Essential services
Real Estate Activities	164,030	0	Other
Professional, Scientific And Technical Activities	1,516,450	1,438,350	Essential services
Administrative And Support Services Activities	1,027,920	662,850	Other
Public Administration And Defence; Compulsory Social Security	1,242,610	1,242,610	Essential services
Education	1,589,450	1,589,450	Essential services
Human Health And Social Work Activities	1,922,250	1,922,250	Health care
Arts, Entertainment And Recreation	318,180	0	Other
Other Services Activities	711,620	280,760	Other
Activities of Households as Employers; Undifferentiated Goods and Services Producing Activities of Households for Own Use	738,910	733,340	Health care

Model initialization

The model population was initialized using age structure data by the Italian National Institute of Statistics [16] and distributed across employment sectors using age-specific data from before lockdown [4]; the remaining population in each age class was assigned to the class of inactive people (unemployed, not gainfully occupied - including children, retired individuals). The population was assumed to be fully susceptible, except for a number N_i of initially infectious individuals on February 1, 2020, who are uniformly distributed across the three stages of infection I, J, K. We neglect population immunity due to transmissions occurring before February 1. N_i was a free parameter estimated during the calibration procedure.

Model output

The main output of the model is the age-specific number of new infections per day, $i_a(t)$. From these, we compute the age-specific number of symptomatic infections $s_a(t)$ by applying an age-specific probability of respiratory symptoms σ_a estimated from contact tracing data in Lombardy ([21], reported in Table S4). The total daily number of cases admitted to the hospital, $H(t)$, is computed as a fixed proportion h of the total number of symptomatic cases, delayed by the average time between symptom onset and hospital admission, τ_H, so that:

$$H(t) = h\, s_a(t-\tau_H) = h \sum_a \sigma_a i_a(t-\tau_h)$$

The total daily number of critical patients admitted to an ICU, $Q(t)$, is computed as a fraction q of the total hospitalized cases, delayed by the average time τ_Q between hospital admission and ICU admission

$$Q(t) = q\, H(t-\tau_Q).$$

The total occupancy of hospital beds, $B_H(t)$, and of ICU beds, $B_Q(t)$, were estimated by a stochastic implementation of the following differential equation models:

$$B_H{}'(t) = H(t) - \frac{1}{d_H} B_H(t)$$

$$B_Q{}'(t) = Q(t) - \frac{1}{d_Q} B_Q(t)$$

Where d_H and d_Q are the average lengths of stay in hospital and ICU respectively. Parameters q, τ_H, τ_Q, d_H and d_Q were estimated from the analysis of hospital data from over 45,000 COVID-19 patients in Lombardy [22] and are reported in Table S5, while h was a free parameter estimated during model calibration (see below).

Table S4. Probability of respiratory symptoms or fever >37.5°C by age [21].

Age group (years)	Probability (%)
0-19	18.1
20-39	22.4
40-59	30.5
60-79	35.5
80+	64.6
**Overall**	**31.0**

Table S5. Parameters used to estimate quantities related to the health burden of COVID-19 from age-specific infections computed by the dynamic model [22].

Parameter	Symbol	Value	Unit
Proportion of hospitalized patients who require intensive care	q	11.7	%
Average delay between symptom onset and hospitalization	τ_H	7	Days
Average delay between hospitalization and ICU admission	τ_Q	5	Days
Average length of stay in hospital	d_H	16.4	Days
Average length of stay in ICU	d_Q	14.6	Days

Estimate of the stage-specific relative infectiousness χ_I, χ_J and χ_K

We estimated the stage-specific relative infectiousness χ_I, χ_J and χ_K in such a way that the resulting distribution of the generation time reproduces the observed distribution of the serial interval, i.e. a gamma distribution with shape 1.87 and rate 0.28 [9]. To this aim, we built a continuous-time Markov chain process based on four states (the three stages of infectiousness I, J, K and a state representing recovered individuals R) where the transition between stages is assumed to be exponentially distributed with mean γ. Given a basic reproduction number R_o, the time at infection for each secondary case generated in a fully susceptible population by a single index case during each stage is assumed to be exponentially distributed with mean $R_o\gamma\chi_I, R_o\gamma\chi_J, R_o\gamma\chi_K$, respectively. We simulated the time at infection of secondary cases generated by 10,000 distinct index cases in a fully susceptible population with different sets of values for χ_I, χ_J, χ_K and γ and evaluated the obtained distribution of the generation times. The final set of parameter values was selected by minimizing the root-mean-square-error (RMSE) between the modeled distribution of the generation times and the observed distribution of the serial interval, obtaining $\chi_I = 0.042, \chi_J = 2.700$ and $\chi_K = 0.258$ with a $\gamma = 0.303$. This result is consistent with the expectation of a low infectivity in the first days post-exposure (stage I), that the peak of infectiousness occurs in stage J and that infectiousness fades with the declining viral load [23]. A comparison between the resulting distributions of the modeled generation time and the observed SARS-CoV-2 serial interval is shown in Figure S1.

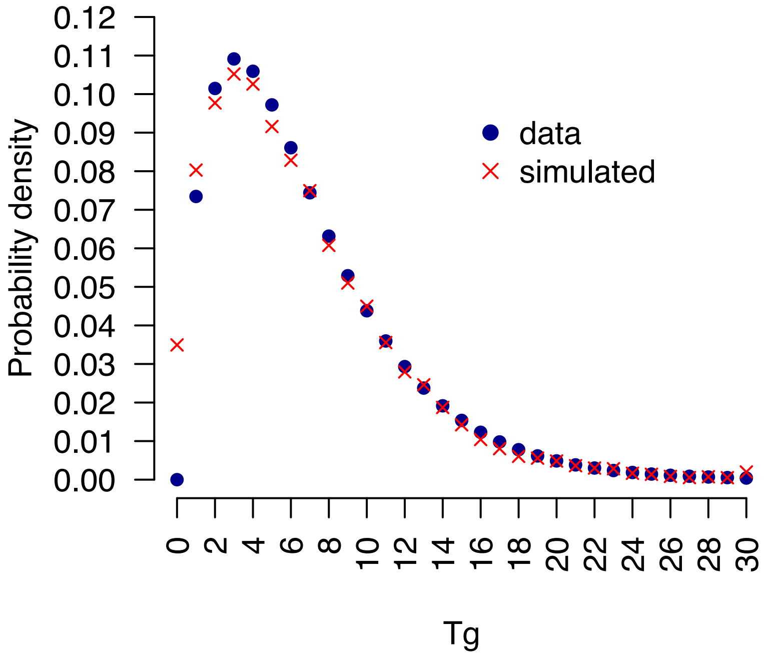

Figure S1. Comparison of the observed distribution of the SARS-CoV-2 serial interval [9] (blue) and the model-simulated distribution of the generation time (red) with γ=0.303 days-1, $\chi_I = 0.042, \chi_J = 2.700, \chi_K = 0.258$. Tg: generation time

Estimate of the scaling factor for transmissibility, β

We analytically computed a distribution for the transmissibility scaling factor, β, such that in absence of interventions the ensuing distribution of the model's basic reproduction number R_0 reproduces a desired distribution. The model's basic reproduction number R_0 can be computed as the dominant eigenvalue of the Next Generation Matrix (NGM) [24] associated with the dynamical system considered. More specifically, in absence of interventions, $\alpha_a^S(t=0) = \alpha_a^T(t=0) = \alpha_a^L(t=0) = \alpha_a^O(t=0) = 1$ and the overall contact matrix at t=0 is:

$$M_{a,\tilde{a}}^e(t=0) = H_{a,\tilde{a}} + S_{a,\tilde{a}} + T_{a,\tilde{a}} + L_{a,\tilde{a}} + O_{a,\tilde{a}} + E_{a,\tilde{a}}^e$$

Thus, the resulting next-generation matrix is given by a block matrix defined as follows:

$$NGM = \begin{pmatrix} R_{a,\tilde{a}}^1 & R_{a,\tilde{a}}^1 & R_{a,\tilde{a}}^1 & R_{a,\tilde{a}}^1 & R_{a,\tilde{a}}^1 & R_{a,\tilde{a}}^1 & R_{a,\tilde{a}}^1 & R_{a,\tilde{a}}^1 \\ R_{a,\tilde{a}}^2 & R_{a,\tilde{a}}^2 & R_{a,\tilde{a}}^2 & R_{a,\tilde{a}}^2 & R_{a,\tilde{a}}^2 & R_{a,\tilde{a}}^2 & R_{a,\tilde{a}}^2 & R_{a,\tilde{a}}^2 \\ R_{a,\tilde{a}}^3 & R_{a,\tilde{a}}^3 & R_{a,\tilde{a}}^3 & R_{a,\tilde{a}}^3 & R_{a,\tilde{a}}^3 & R_{a,\tilde{a}}^3 & R_{a,\tilde{a}}^3 & R_{a,\tilde{a}}^3 \\ R_{a,\tilde{a}}^4 & R_{a,\tilde{a}}^4 & R_{a,\tilde{a}}^4 & R_{a,\tilde{a}}^4 & R_{a,\tilde{a}}^4 & R_{a,\tilde{a}}^4 & R_{a,\tilde{a}}^4 & R_{a,\tilde{a}}^4 \\ R_{a,\tilde{a}}^5 & R_{a,\tilde{a}}^5 & R_{a,\tilde{a}}^5 & R_{a,\tilde{a}}^5 & R_{a,\tilde{a}}^5 & R_{a,\tilde{a}}^5 & R_{a,\tilde{a}}^5 & R_{a,\tilde{a}}^5 \\ R_{a,\tilde{a}}^6 & R_{a,\tilde{a}}^6 & R_{a,\tilde{a}}^6 & R_{a,\tilde{a}}^6 & R_{a,\tilde{a}}^6 & R_{a,\tilde{a}}^6 & R_{a,\tilde{a}}^6 & R_{a,\tilde{a}}^6 \\ R_{a,\tilde{a}}^7 & R_{a,\tilde{a}}^7 & R_{a,\tilde{a}}^7 & R_{a,\tilde{a}}^7 & R_{a,\tilde{a}}^7 & R_{a,\tilde{a}}^7 & R_{a,\tilde{a}}^7 & R_{a,\tilde{a}}^7 \\ R_{a,\tilde{a}}^8 & R_{a,\tilde{a}}^8 & R_{a,\tilde{a}}^8 & R_{a,\tilde{a}}^8 & R_{a,\tilde{a}}^8 & R_{a,\tilde{a}}^8 & R_{a,\tilde{a}}^8 & R_{a,\tilde{a}}^8 \end{pmatrix}$$

where each block is defined as

$$R_{a,\tilde{a}}^e = \beta \frac{\chi_I + \chi_J + \chi_K}{\gamma} r_a M_{a,\tilde{a}}^e \frac{N_a^e}{\sum_c N_{\tilde{a}}^c}$$

with N_a^e representing the number of individuals of age a in the employment group e.

The resulting reproduction number is provided by $R_0 = s(NGM)$, where $s(NGM)$ is the spectral radius of NGM (i.e., the largest absolute value of its eigenvalues). Thus, the distribution of β can be computed analytically from the desired distribution of R_0, given the distribution of the age-specific susceptibility profile, r_a and on the distribution of the bootstrapped contact matrix.

We compute individual samples of the distribution of β by iteratively choosing one sample from the distribution of the desired R_0, one sample from the known distribution of the age-specific susceptibility [11], and one sample from the bootstrapped contact matrices. At the end of this procedure, we obtain a joint distribution of $\{\beta, r_a, C_{a,\tilde{a}}\}$. We considered estimates of R_0 computed from the curve of symptomatic cases by date of symptom onset using the method reported in [9, 10, 25] (mean estimate 2.99, 95%CI 2.88-3.11), which resulted in a mean value of β equal to 0.0148 (95%CI 0.0126-0.0167). The distribution of β was re-estimated with the same approach for alternative models in the sensitivity analysis and for regions in the subnational analysis (see below).

Model calibration

We calibrated model parameters against the observed daily curve of hospitalized cases up to September 30, obtained from surveillance data, using a Markov Chain Monte Carlo (MCMC) approach with Poisson likelihood with reversible normal jumps and a Metropolis-Hastings acceptance algorithm. Free model parameters were:

- the number of infectious individuals at February 1, N_i;
- the reduction in the per-contact transmission rate between the first detection of local transmission in Italy (February 21) and the first reopening after the lockdown phase (May 4 in the actual scenario, used for calibration) φ_1;
- the reduction in the per-contact transmission rate between the first reopening after the lockdown phase (May 4) and the end of simulations, φ_2;
- the average proportion of hospital admissions for cases with respiratory symptoms, h.

Uninformative (uniform) priors were assumed for N_i, φ_1 and φ_2, but we restricted N_i to be strictly positive and φ_1 and φ_2 to be bounded between 0 and 1. For h, we assumed a uniform prior distribution limited by two boundary values identified from estimates of the average proportion of infections which result in respiratory symptoms, $\bar{\sigma}$, the mortality rate observed for hospitalized individuals, μ_H, and the SARS-CoV-2 infection fatality rate IFR. In particular, the following relation holds:

$$IFR = \bar{\sigma}\, h\, \mu_H,$$

from which follows

$$h = \frac{IFR}{\bar{\sigma}\, \mu_H}$$

Considering $\bar{\sigma}$ = 31.0% [21] (Table S4), μ_H= 27.6% (95%CI: 27.4-27.8%) [22], and that the large majority of IFR estimates have been reported to be between 0.5 and 2%, [26], we obtain that h must be bounded between 5% and 25%.

At each step of the MCMC, a new sample from the joint distribution of $\{\beta, r_a, C_{a,\tilde{a}}\}$ is considered, together with a new proposal for the free model parameters. The posterior joint distribution of $\{\beta, r_a, C_{a,\tilde{a}}\}$ resulted to be identical to the prior, indicating that the MCMC does not apply any selection on these parameters. Table S6 reports a summary of the estimated posterior distributions of free parameters. Epidemiological results were obtained by running one stochastic simulation for 10,000 of the last 30,000 parameter sets accepted in the MCMC (including the corresponding samples from $\{\beta, r_a, C_{a,\tilde{a}}\}$).

Table S6. Posterior values for the estimated parameters

Parameter	Mean	95%CI
$\boldsymbol{N_i}$, number of infectious individuals at Feb 1	1985	688-3806
$\boldsymbol{\varphi_1}$, reduction in transmission efficiency Feb 21 - May 3	0.30	0.14-0.43
$\boldsymbol{\varphi_2}$, reduction in transmission efficiency May 4 - Jul 10	0.44	0.36-0.52
$\boldsymbol{h}$, average proportion of hospital admissions for cases with respiratory symptoms	0.135	0.055-0.239

Additional results on the baseline model

The model estimated an overall attack rate of 4.8% (95%CI: 2.0-10.5%) in the Italian population on September 30 (Figure S2), corresponding to approximately 2.9 million infections (95%CI: 1.2-6.3 million). The age-profile of the attack rate reproduces qualitatively well the findings of a large-scale seroprevalence study in Spain, identifying lowest attack rates in the under 19 population, followed by the population above 65, and roughly constant values for adults of intermediate age. Table S7 reports estimates of the case ascertainment ratios, disaggregated by period (until June 30 and between July 1 and September 30) and by symptomatic status, computed as the ratio of the number of ascertained cases and the corresponding model estimated infections.

Table S7. Number of ascertained cases, model-estimated infections and estimated case ascertainment ratios before and after June 30, 2020

	Until June 30, 2020		July 1 – September 30, 2020	
	Value or mean	95%CI	Value or mean	95%CI
Cases ascertained (data from the national surveillance system)				
Total	241,542	-	76,970	-
Symptomatic	155,537	-	30,364	-
Asymptomatic	86,005	-	46,606	-
Estimated infections				
Total	2,571,336	1,080,444-5,613,480	313,872	132,792-730,356
Symptomatic	797,114	334,938-1,740,179	97,300	41,166-226,410
Asymptomatic	1,774,222	745,506-3,873,301	216,572	91,626-503,946
Estimated case ascertainment ratio				
Total	9.4%	4.3-22.4%	24.5%	10.5-58.0%
Symptomatic	19.5%	8.94-46.4%	31.2%	13.4-73.8%
Asymptomatic	4.8%	2.2-11.5%	21.5%	9.2-50.9%

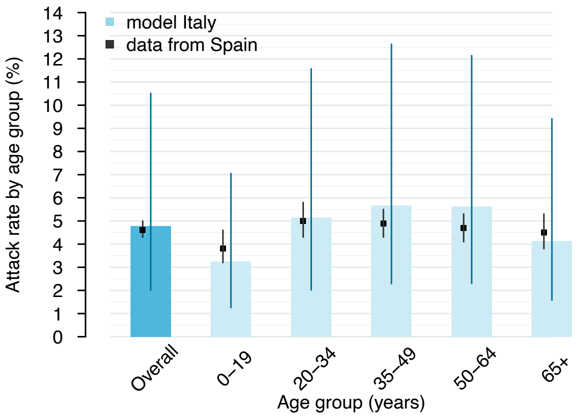

Figure S2. Model-estimated attack rate as of September 30, in the overall population and by age classes, and comparison with corresponding age-specific data from a large scale seroprevalence study in Spain [13].

Figure S3 reports the daily COVID-19 hospitalizations estimated by the model in all considered scenarios (see Figure 2E and Table 1 in the main text for a description of scenarios).

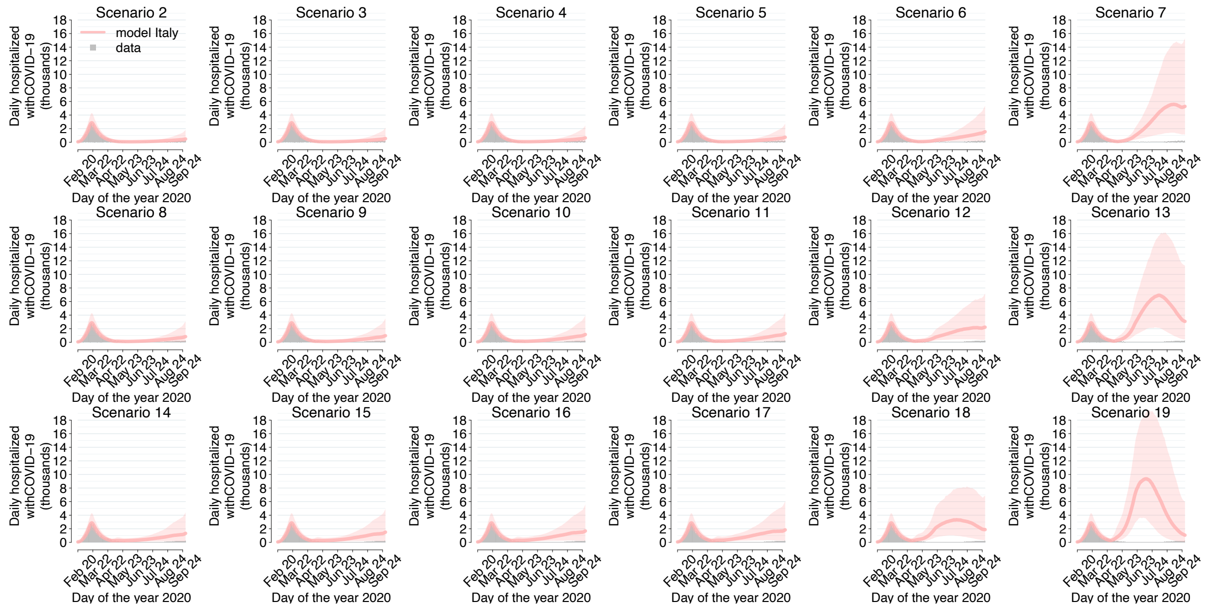

Figure S3. Daily number of hospitalized patients with COVID-19 in the counterfactual scenarios

Sensitivity analyses

Despite fast-paced progress in an extremely short time since the start of the epidemics, there are still many unknowns in the epidemiology of COVID-19. For this reason, we tested the sensitivity of our results by repeating the same analyses using a number of alternative models encoding different epidemiological assumptions. In particular, we considering the following alternative variations on the baseline model:

- Sensitivity A considers that individuals have the same susceptibility $r_a = 1$, independently of their age;
- Sensitivity B considers the possibility that children below 15 years old may be less efficient in transmitting infection (50% compared to adults); formally, the scaling factor shaping the transmission rate is considered to be age-dependent, β_a, taking values 0.5 β for $a < 15$ and β for all other ages;
- Sensitivity C considers the same integrated occupational risk of infection across all employment sectors, setting $\rho_\varepsilon = 1$ for all ε in Eq. 3;
- Sensitivity D considers integrated occupational risks ρ_ε equal to the baseline, but with halved risks for healthcare workers and a doubled risk for employees of the manufacturing and construction sectors.

In all models, we re-computed the distribution of parameter β (Table S8) and then recalibrated the free model parameters using the same methods described above for the baseline model. Figure S4 compares the posterior distribution of parameters in the different models, showing largely consistent estimates with respect to the baseline. Only for model A we estimate a slightly lower reduction in transmissibility after May 4. Figure S5 shows that all models were able to correctly fit hospital admission data and Figure S6 compares the cumulative number of hospitalizations under actual interventions across all models. Tables S9-S12 provide results obtained with models A-D, corresponding to those of the baseline model reported in Table 1 in the main text. Figure S7 shows the age profile of attack rates at September 30 in the actual interventions (scenario 1), estimated by the different models considered in the sensitivity analysis. Only model A (homogeneous susceptibility across ages) estimates a qualitatively different profile from all other models (including the baseline), suggesting an attack rate in age-class 0-19 as high as that of individuals 35-64, and a very low attack rate in the elderly.

Model parameters, daily incidence of hospitalized cases and attack rates by age as estimated by sensitivity models A-D considering governmental interventions (scenario 1) are consistent with those estimated by the baseline model. The only exception is represented by the higher attack rate in age class 0-19 years estimated by model A (average 5.7%, compared to the approximately 3.5% estimated in all other models), determined by the assumption of equal susceptibility to infection by age.

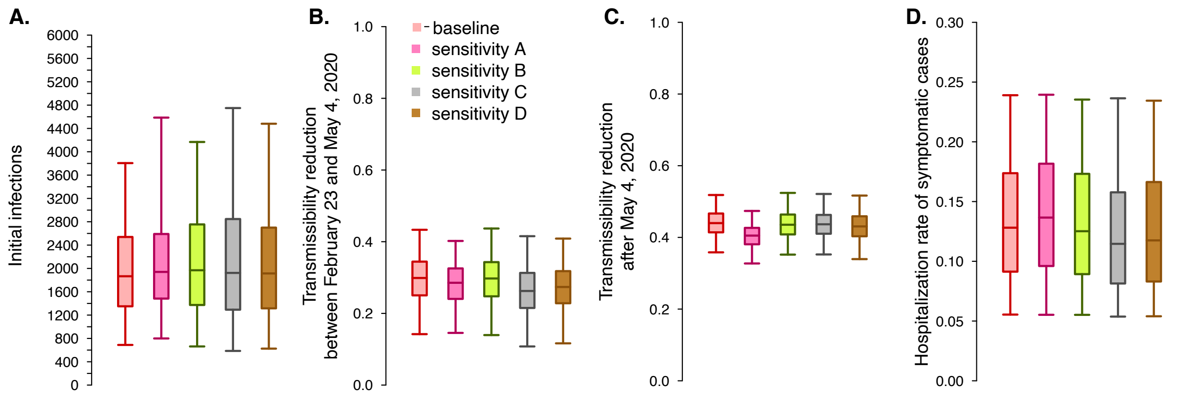

Figure S4. Comparison of posterior estimates for free model parameters. Boxplots represent the mean of the posterior distribution, the interquartile ranges, and the 95% credible intervals.

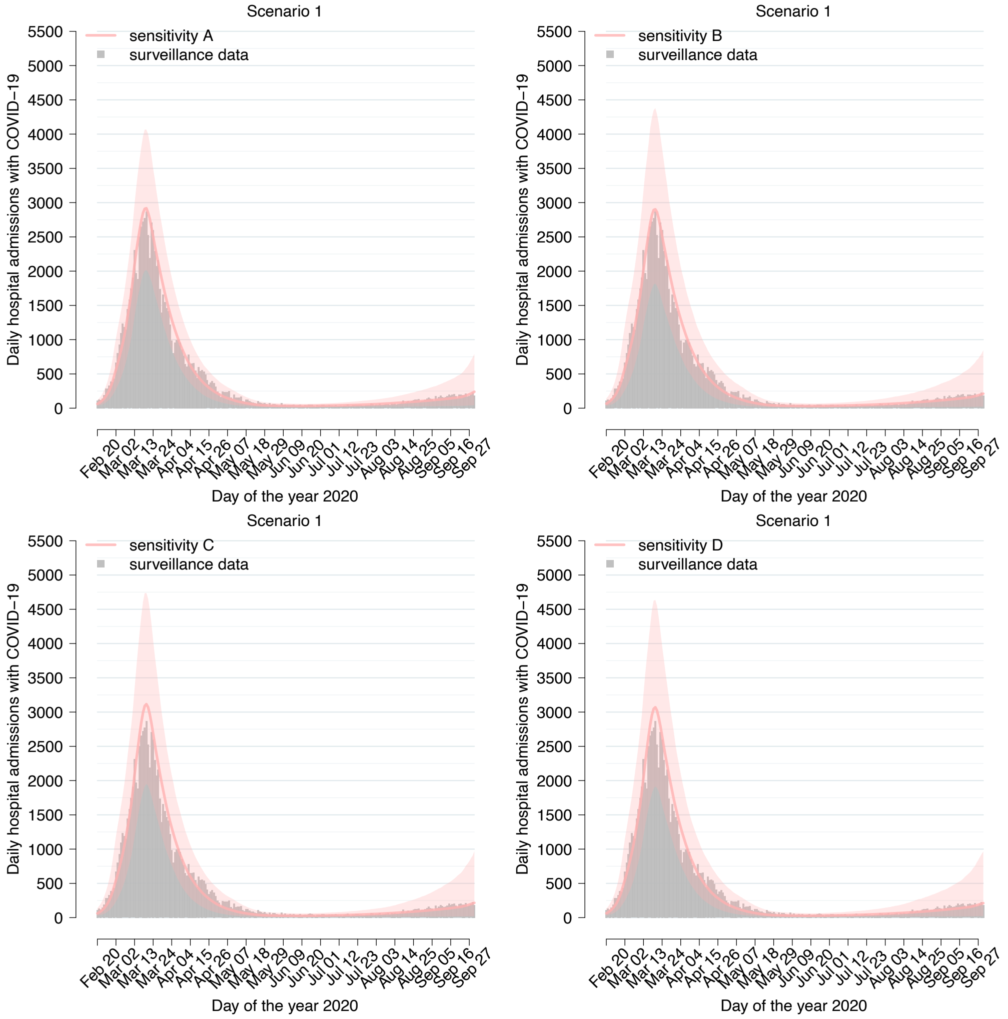

Figure S5. Fit of hospital admission data with the different sensitivity analyses

Table S8. Summary of the computed distributions of β (mean and 95%CI) for the different sensitivity analyses.

Sensitivity	β
A	0.0137 (0.0124-0.0148)
B	0.0150 (0.0134-0.0165)
C	0.0148 (0.0133-0.0162)
D	0.0148 (0.0133-0.0162)

Table S9. Characteristics of considered scenarios and simulation results under Sensitivity A.

Scenario ID	Reopening of selected productive sectors	Lifting of lockdown	Schools reopened*	Complete reopening	Cumulative hospitalized cases	Cumulative ICU cases	Hospital bed occupancy at September 30	ICU bed occupancy at September 30
**Observed**	May 4	May 18	-	No	95,076	-	3,327	280
**1 (actual interventions)**	May 4	May 18	-	No	96,032 [69,686-129,441]	11,080 [8,017-14,924]	2,615 [572-7,859]	241 [54-694]
**2**	-	May 4	-	No	111,066 [79,084-157,119]	12,661 [9,037-17,728]	6,331 [1,339-18,282]	600 [133-1,672]
**3**	-	May 4	K	No	118,380 [83,127-172,423]	13,423 [9,495-19,196]	8,491 [1,747-23,991]	806 [175-22,14]
**4**	-	May 4	KP	No	133,360 [90,167-202,617]	15,004 [10,259-22,295]	12,310 [2,768-33,996]	1,171 [274-3,147]
**5**	-	May 4	KPS	No	167,554 [104,492-278,066]	18,737 [11,987-30,000]	20,401 [4,824-55,176]	1,979 [481-5,243]
**6**	-	May 4	KPSH	No	229,666 [129,185-406,347]	25,649 [14,686-44,383]	32,953 [8,424-84,529]	3,298 [864-8,249]
**7**	-	May 4	KPSH	Yes	625,071 [309,371-1,127,296]	70,024 [34,784-125,297]	91,902 [23,343-218,535]	9,866 [2,607-22,963]
**8**	-	April 27	-	No	133,392 [90,835-200,602]	15,039 [10,361-22,236]	11,494 [2,560-31,821]	1,102 [254-2,985]
**9**	-	April 27	K	No	147,015 [97,785-230,000]	16,544 [11,183-25,395]	15,525 [3,435-40,502]	1,486 [339-3,851]
**10**	-	April 27	KP	No	180,046 [111,136-296,187]	20,080 [12,685-32,282]	22,389 [5,635-58,054]	2,191 [564-5,510]
**11**	-	April 27	KPS	No	256,338 [144,829-451,772]	28,586 [16,367-49,727]	35,563 [9,608-89,853]	3,571 [1,012-8,843]
**12**	-	April 27	KPSH	No	375,747 [205,178-676,523]	42,160 [23,342-75,655]	46,037 [13,194-117,043]	4,845 [1,432-12,060]
**13**	-	April 27	KPSH	Yes	799,691 [406,113-1,385,228]	92,512 [47,111-160,385]	58,131 [9,377-171,236]	6,589 [1,063-18,847]
**14**	-	April 20	-	No	172,373 [110,193-273,292]	19,300 [12,642-30,157]	19,243 [4,735-50,408]	1,893 [478-4,817]
**15**	-	April 20	K	No	198,659 [121,957-322,795]	22,197 [13,904-35,716]	25,214 [6,390-63,489]	2,496 [639-6,147]
**16**	-	April 20	KP	No	257,801 [148,119-437,192]	28,896 [16,984-48,600]	33,765 [9,216-84,318]	3,447 [967-8,415]
**17**	-	April 20	KPS	No	385,575 [217,243-692,885]	43,453 [24,777-77,690]	43,658 [11,670-111,939]	4,597 [1,286-11,716]
**18**	-	April 20	KPSH	No	557,183 [302,975-972,625]	63,813 [35,127-111,530]	38,212 [7,104-109,088]	4,253 [813-11,894]
**19**	-	April 20	KPSH	Yes	949,047 [470,001-1,610,584]	110,370 [54,922-186,529]	23,721 [3,145-92,817]	2,709 [338-10,611]

*: K: kindergartens; P: primary; S: secondary; H: high schools. Reopening is assumed on the same day the lockdown is lifted

Table S10. Characteristics of considered scenarios and simulation results under Sensitivity B.

Scenario ID	Reopening of selected productive sectors	Lifting of lockdown	Schools reopened *	Complete reopening	Cumulative hospitalized cases	Cumulative ICU cases	Hospital bed occupancy at September 30	ICU bed occupancy at September 30
**Observed**	May 4	May 18	-	No	95,076	-	3,327	280
**1 (actual interventions)**	May 4	May 18	-	No	96,534 [64,020-143,093]	11,133 [7,357-16,493]	2,501 [343-8,867]	236 [34-803]
**2**	-	May 4	-	No	111,382 [72,526-174,048]	12,728 [8,324-19,544]	6,215 [832-21,618]	599 [86-2,001]
**3**	-	May 4	K	No	114,550 [74,189-178,848]	13,036 [8,528-20,111]	6,999 [979-24,405]	679 [99-2,290]
**4**	-	May 4	KP	No	118,905 [76,508-187,224]	13,509 [8,769-21,033]	8,058 [1,130-27,785]	785 [115-2,603]
**5**	-	May 4	KPS	No	121,839 [77,948-196,155]	13,879 [8,936-21,852]	8,948 [1,263-30,763]	868 [129-2,891]
**6**	-	May 4	KPSH	No	171,854 [94,071-323,397]	19,192 [10,799-35,176]	20,552 [3,123-65,070]	2,060 [316-6,387]
**7**	-	May 4	KPSH	Yes	507,021 [213,909-1,022,185]	56,146 [23,718-112,697]	86,413 [24,513-229,819]	9,122 [2,596-24,179]
**8**	-	April 27	-	No	134,618 [83,625-221,699]	15,255 [9,579-24,511]	11,363 [1,612-37,172]	1,115 [171-3,566]
**9**	-	April 27	K	No	140,639 [85,264-238,430]	15,871 [9,817-26,032]	12,853 [1,878-42,082]	1,261 [194-3,980]
**10**	-	April 27	KP	No	150,032 [88,785-260,067]	16,827 [10,268-28,516]	15,011 [2,317-48,099]	1,484 [236-4,638]
**11**	-	April 27	KPS	No	157,625 [91,037-278,152]	17,728 [10,500-30,545]	16,648 [2,565-51,729]	1,650 [265-5,060]
**12**	-	April 27	KPSH	No	271,576 [134,481-531,778]	30,461 [15,352-59,196]	35,300 [7,069-100,797]	3,642 [734-10,404]
**13**	-	April 27	KPSH	Yes	664,691 [328,433-1,300,913]	75,877 [37,062-149,081]	66,988 [15,781-196,400]	7,498 [1,789-21,553]
**14**	-	April 20	-	No	176,364 [98,915-310,000]	19,891 [11,375-34,213]	19,129 [3,103-57,740]	1,917 [321-5,702]
**15**	-	April 20	K	No	187,569 [103,229-334,352]	21,069 [11,778-36,942]	21,374 [3,648-63,931]	2,166 [379-6,349]
**16**	-	April 20	KP	No	205,241 [111,295-372,398]	23,098 [12,608-40,707]	24,206 [4,280-72,071]	2,469 [442-7,217]
**17**	-	April 20	KPS	No	220,078 [115,464-402,571]	24,655 [13,222-44,083]	26,135 [4,827-78,634]	2,683 [508-7,873]
**18**	-	April 20	KPSH	No	409,580 [203,026-806,956]	46,582 [23,139-90,501]	38,140 [9,281-115,504]	4,174 [1,019-12,344]
**19**	-	April 20	KPSH	Yes	797,933 [411,204-1,511,256]	92,415 [47,841-175,026]	34,876 [6,158-134,303]	3,983 [684-15,229]

*: K: kindergartens; P: primary; S: secondary; H: high schools. Reopening is assumed on the same day the lockdown is lifted

Table S11. Characteristics of considered scenarios and simulation results under Sensitivity C.

Scenario ID	Reopening of selected productive sectors	Lifting of lockdown	Schools reopened *	Complete reopening	Cumulative hospitalized cases	Cumulative ICU cases	Hospital bed occupancy at September 30	ICU bed occupancy at September 30
**Observed**	May 4	May 18	-	No	95,076	-	3,327	280
**1 (actual interventions)**	May 4	May 18	-	No	97,110 [63,062-143,546]	11,205 [7,300-16,492]	2,479 [294-9,892]	233 [28-875]
**2**	-	May 4	-	No	111,514 [71,554-173,464]	12,713 [8,218-19,468]	6,088 [704-23,375]	583 [71-2,141]
**3**	-	May 4	K	No	114,965 [73,347-182,725]	13,085 [8,411-20,264]	7,087 [776-26,797]	682 [80-2,504]
**4**	-	May 4	KP	No	119,664 [75,795-197,995]	13,599 [8,694-21,801]	8,339 [951-31,268]	802 [101-2,932]
**5**	-	May 4	KPS	No	124,797 [77,258-206,889]	14,101 [8,867-22,904]	9,573 [1,118-35,873]	930 [112-3,374]
**6**	-	May 4	KPSH	No	172,867 [95,915-335,104]	19,245 [10,857-36,544]	21,149 [2,716-72,811]	2,088 [273-7,048]
**7**	-	May 4	KPSH	Yes	495,277 [206,187-1,049,989]	54,627 [22,845-115,084]	90,398 [21,571-248,527]	9,441 [2,222-25,573]
**8**	-	April 27	-	No	134,831 [82,096-230,377]	15,232 [9,394-25,266]	11,582 [1,371-40,984]	1,127 [142-3,815]
**9**	-	April 27	K	No	142,218 [84,342-244,581]	15,973 [9,638-27,028]	13,478 [1,608-46,136]	1,318 [168-4,369]
**10**	-	April 27	KP	No	153,410 [88,764-272,002]	17,212 [10,100-29,593]	16,133 [2,019-54,085]	1,581 [206-5,154]
**11**	-	April 27	KPS	No	165,090 [92,366-298,173]	18,447 [10,534-32,667]	18,573 [2,419-61,672]	1,833 [247-5,944]
**12**	-	April 27	KPSH	No	282,481 [136,302-576,109]	31,441 [15,612-62,757]	39,038 [6,704-115,820]	4,014 [711-11,849]
**13**	-	April 27	KPSH	Yes	673,846 [319,209-1,368,605]	76,937 [36,158-156,290]	71,984 [17,023-222,645]	8,043 [1,908-24,533]
**14**	-	April 20	-	No	180,117 [99,429-325,131]	20,210 [11,384-35,496]	20,485 [2,737-65,695]	2,044 [288-6,428]
**15**	-	April 20	K	No	193,447 [105,111-354,258]	21,617 [11,854-38,967]	23,394 [3,369-72,476]	2,351 [347-7,169]
**16**	-	April 20	KP	No	216,605 [113,114-405,273]	24,177 [12,980-44,465]	27,079 [4,198-84,553]	2,767 [435-8,347]
**17**	-	April 20	KPS	No	238,663 [123,492-455,063]	26,592 [13,919-50,186]	30,649 [4,930-93,670]	3,128 [513-9,332]
**18**	-	April 20	KPSH	No	440,662 [207,762-899,688]	50,215 [23,803-101,458]	43,985 [9,350-133,029]	4,798 [1,028-14,328]
**19**	-	April 20	KPSH	Yes	810,261 [408,823-1,619,262]	94,337 [47,473-187,981]	34,947 [6,334-14,6934]	3,987 [717-16,733]

*: K: kindergartens; P: primary; S: secondary; H: high schools. Reopening is assumed on the same day the lockdown is lifted

Table S12. Characteristics of considered scenarios and simulation results under sensitivity D.

Scenario ID	Reopening of selected productive sectors	Lifting of lockdown	Schools reopened *	Complete reopening	Cumulative hospitalized cases	Cumulative ICU cases	Hospital bed occupancy at September 30	ICU bed occupancy at September 30
**Observed**	May 4	May 18	-	No	95,076	-	3,327	280
**1 (actual interventions)**	May 4	May 18	-	No	97,155 [64,078-147,369]	11,186 [7,444-16,853]	2,455 [331-9,927]	231 [33-876]
**2**	-	May 4	-	No	111,955 [72,363-182,300]	12,801 [8,303-20,647]	6,072 [807-24,061]	586 [84-2,233]
**3**	-	May 4	K	No	115,609 [74,605-193,684]	13,163 [8,538-21,433]	7,105 [916-27,723]	684 [94-2,579]
**4**	-	May 4	KP	No	120,722 [77,672-205,200]	13,723 [8,856-22,806]	8,381 [1,122-32,071]	811 [114-3,002]
**5**	-	May 4	KPS	No	125,614 [79,653-216,485]	14,242 [9,132-24,116]	9,794 [1,294-38,073]	942 [132-3,538]
**6**	-	May 4	KPSH	No	177,554 [97,903-360,268]	19,724 [11,179-39,277]	22,518 [3,090-72,266]	2,215 [318-7,085]
**7**	-	May 4	KPSH	Yes	560,558 [235,856-1,170,566]	62,398 [26,339-127,348]	97,346 [25,378-257,029]	10,290 [2,688-27,110]
**8**	-	April 27	-	No	136,025 [83,924-240,347]	15,366 [9,655-26,288]	11,603 [1,560-41,534]	1,131 [162-3,937]
**9**	-	April 27	K	No	143,094 [86,444-261,896]	16,125 [9,913-28,597]	13,595 [1,856-47,980]	1,331 [193-4,535]
**10**	-	April 27	KP	No	154,836 [91,224-293,619]	17,353 [10,457-32,145]	16,256 [2,329-54,082]	1,591 [238-5,273]
**11**	-	April 27	KPS	No	166,871 [95,031-323,694]	18,641 [10,997-35,556]	18,964 [2,740-61,586]	1,870 [283-6,022]
**12**	-	April 27	KPSH	No	286,659 [139,301-582,871]	32,045 [15,824-64,495]	38,663 [7,302-112,929]	4,011 [768-11,480]
**13**	-	April 27	KPSH	Yes	728,103 [355,551-1,444,384]	83,198 [40,558-16,5153]	70,048 [15,272-218,818]	7,910 [1,749-24,251]
**14**	-	April 20	-	No	180,404 [103,137-338,100]	20,405 [11,727-37,025]	20,429 [3,051-63,419]	2,044 [315-6,327]
**15**	-	April 20	K	No	193,876 [108,177-366,504]	21,750 [12,319-40,276]	23,251 [3,626-70,585]	2,339 [382-6,970]
**16**	-	April 20	KP	No	217,129 [114,847-416,655]	24,324 [13,202-46,110]	27,018 [4,396-81,658]	2,754 [467-8,096]
**17**	-	April 20	KPS	No	239,282 [122,687-460,324]	26,801 [14,079-51,577]	30,260 [5,320-89,462]	3,105 [570-9,042]
**18**	-	April 20	KPSH	No	441,083 [213,405-891,098]	50,155 [24,346-101,116]	42,380 [9,412-125,586]	4,650 [1,031-13,324]
**19**	-	April 20	KPSH	Yes	846,292 [427,906-1,653,881]	98,215 [49,853-191,376]	32,972 [5,557-138,600]	3,772 [620-15,755]

*: K: kindergartens; P: primary; S: secondary; H: high schools. Reopening is assumed on the same day the lockdown is lifted

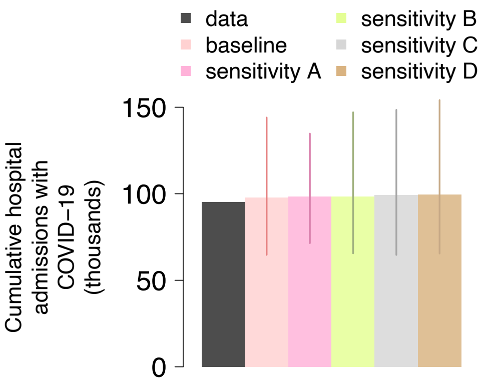

Figure S6. Comparison of model estimates on the cumulative number of hospitalizations in the actual interventions (scenario 1).

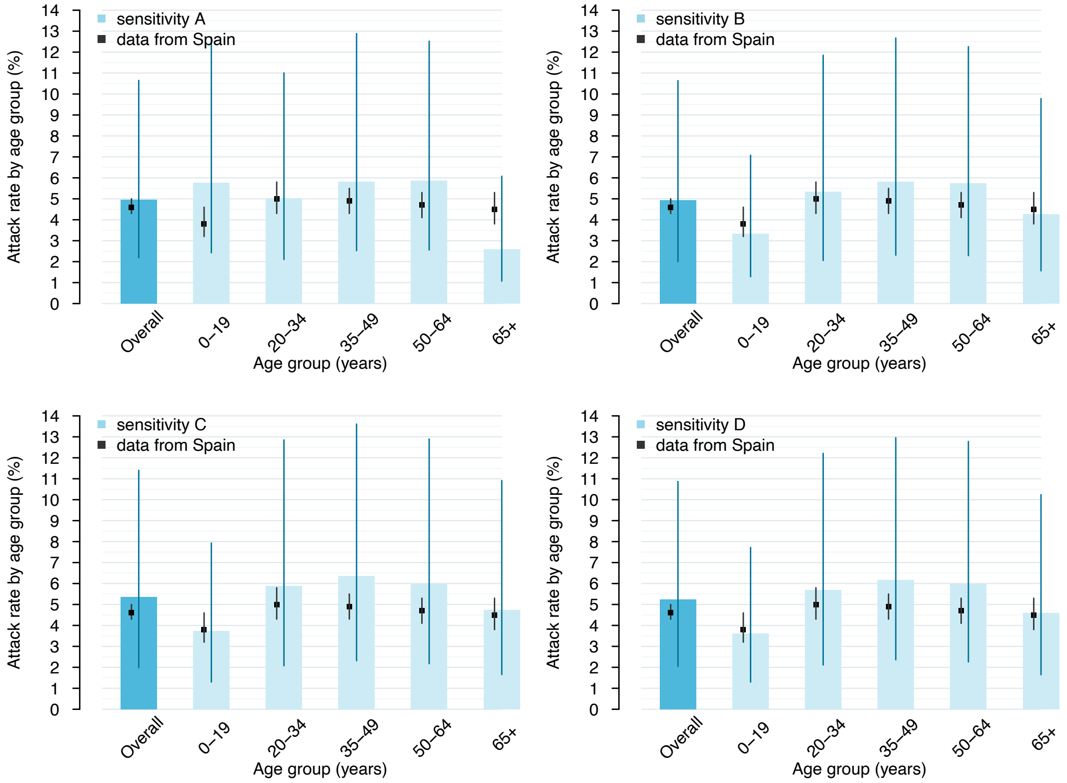

Figure S7. Attack rate as of September 30, estimated by the different models considered in the sensitivity analysis in the overall population and by age classes; corresponding age-specific data from a large scale seroprevalence study in Spain are reported for comparison [13].

Subnational analyses

The model and calibration procedures were applied to three regions representative of Southern (Campania), Central (Lazio) and Northern Italy (Lombardy), adjusting relevant quantities to the specific geographic context. In particular, we considered regional estimates for the distribution of the basic reproduction number [25] (Table S13) and the regional demographic structure (Figure S8) to compute a region-specific distribution for β (Table S14). Then, the four free model parameters were re-calibrated on the regional curve of daily hospital admissions with COVID-19. Because of the likely later timing of introduction of SARS-CoV-2 in Lazio and Campania, we considered February 13 instead of February 1 as the date at which the initial number of infectious individuals is estimated.

Table S13. Estimates of regional R_0 [25] and corresponding computed distributions for β (mean and 95%CI).

Region	R_0	β
Campania	2.86 (2.36-3.45)	0.0142 (0.0117-0.0175)
Lazio	2.89 (2.55-3.26)	0.0143 (0.0122-0.0165)
Lombardy	2.93 (2.74-3.14)	0.0145 (0.0129-0.0163)

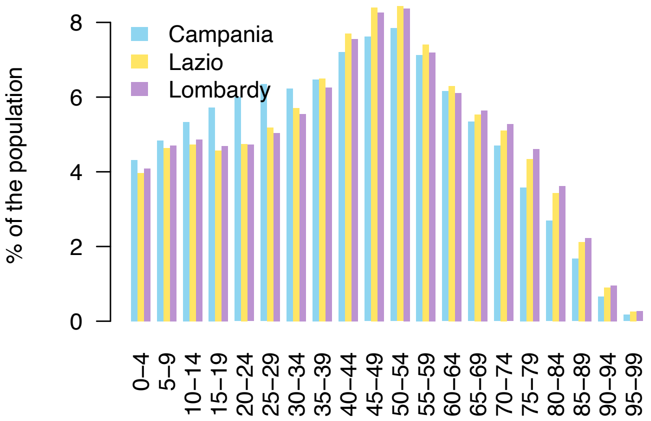

Figure S8. Comparison of population age structures across selected regions (data from [16]).

Table S14 and Figure S9 show similar estimates for the estimated posterior distribution of free parameter values across regions, except for the number of initially infected individuals. Compared to the corresponding estimates for Italy, the model associates a slightly lower transmissibility reduction after the lifting of lockdown in the three regions, and a slightly higher mean hospitalization rate of symptomatic individuals in Lombardy, but with broadly overlapping confidence intervals. Figures S10-12 show the good agreement between the calibrated model and the observed hospitalization curves.

Table S14. Posterior estimates for region-specific free model parameters.

	Campania		Lazio		Lombardy	
**Parameter**	*Mean*	*95%CI*	*Mean*	*95%CI*	*Mean*	*95%CI*
N_i, number of initial infectious individuals	639	87-2092	849	156-2097	1202	467-2504
φ_1, reduction in transmission efficiency Feb 21 - May 3	0.31	0.08-0.54	0.25	0.05-0.46	0.27	0.11-0.41
φ_2, reduction in transmission efficiency May 4 - Sep 30	0.39	0.22-0.53	0.40	0.30-0.51	0.36	0.20-0.48
h, average proportion of hospital admissions for cases with respiratory symptoms	0.128	0.053-0.238	0.129	0.054-0.239	0.151	0.063-0.241

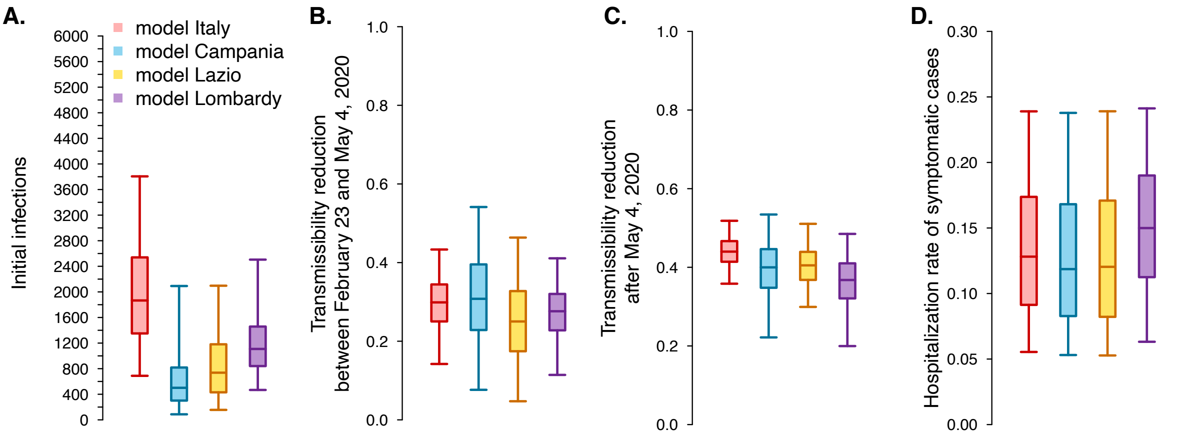

Figure S9. Posterior distributions of parameter values obtained in the subnational analyses compared with corresponding national estimates

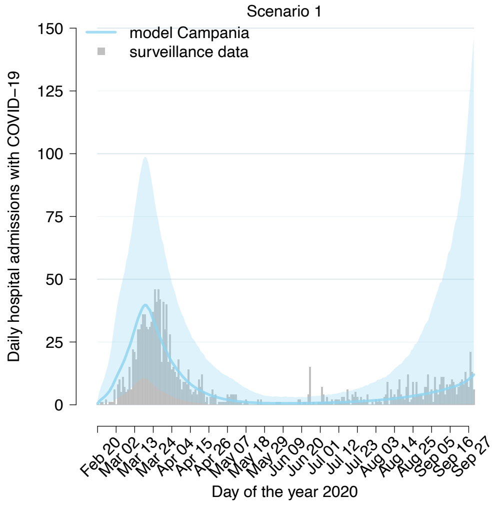

Figure S10. Model fit for hospital admission data in Campania

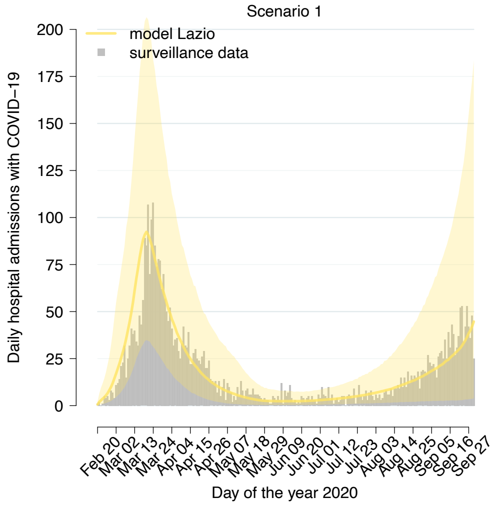

Figure S11. Model fit for hospital admission data in Lazio

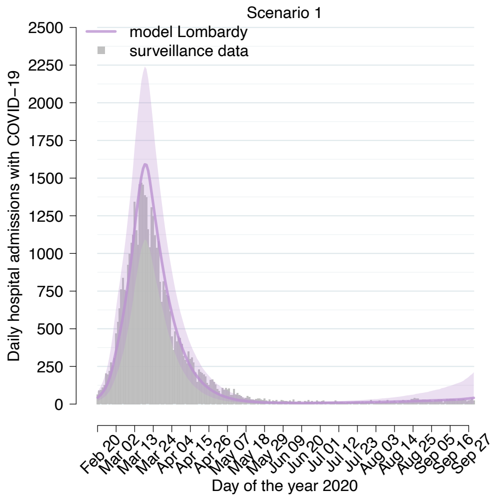

Figure S12. Model fit for hospital admission data in Lombardy

Computation of the net reproduction number

The distribution of the net reproductive number R(t) shown in Figure 2B in the main text was estimated from the time series of observed and modeled cases by applying a well-established statistical method [27, 28]. The method is based on the estimation of the posterior distribution of R for any time point t, by applying the Metropolis-Hastings MCMC sampling to a likelihood function defined as follows:

$$[\text{Eq9}] \quad \mathcal{L} = \prod_{t=1}^{T} P\left(C(t);\ R_t \sum_{s=1}^{T} \psi(s) C(t-s) \right)$$

where

- $P(k; \lambda)$ is the probability mass function of a Poisson distribution (i.e., the probability of observing k events if these events occur with rate λ).
- R_t is the net reproduction number at time t to be estimated;
- $\psi(s)$ is the distribution of the generation time calculated at time s, once again assumed to be approximated by the distribution of the serial interval, i.e. a gamma with shape 1.87 and rate 0.28 (estimated in [9] and shown in Figure S1);
- C(t), is the daily number of new cases at time t.

To compute R_t from observed cases, we used as C(t) the daily number of symptomatic cases by date of symptom onset as recorded in the national surveillance system; for the estimate of R_t from model simulations, we computed the 10,000 C(t) curves from as many model simulations by applying the age-specific probability of symptoms (Table S4) to the modeled age-specific daily infections at their entrance in compartment K (assumed to be the date of symptom onset). The posterior distributions of the estimated R_t from the 10,000 simulations were then pooled together to obtain the overall distribution of R_t from model simulations. For each daily estimate of R_t, we report in Figure 2B in the main text the mean and confidence interval of the posterior distributions (note that for the curve of R_t from observed data, confidence intervals are very narrow around the mean estimate).

References

1. Mossong J, Hens N, Jit M, Beutels P, Auranen K, Mikolajczyk R, Massari M, Salmaso S, Scalia Tomba G, Wallinga J, Heijne J. Social contacts and mixing patterns relevant to the spread of infectious diseases. PLoS Medicine. 2008;5(3).
2. Google. COVID-19 Community Mobility Reports. https://www.google.com/covid19/mobility/
3. Istituto Nazionale di Statistica. Multiscopo sulle famiglie: uso del tempo. https://www.istat.it/it/archivio/216733
4. Italian Institute of Statistics (ISTAT), 2020. Memoria scritta dell'Istituto nazionale di statistica per la 5a Commissione programmazione economica e bilancio del Senato della Repubblica. 25 marzo 2020.

5. Ministero per la Pubblica Amministrazione, Dipartimento Funzione Pubblica. PA: lo smart working nelle Regioni ecco I dati. http://www.funzionepubblica.gov.it/articolo/ministro/25-03-2020/pa-lo-smart-working-nelle-regioni-ecco-i-primi-dati last access April 28, 2020
6. Italian Workers' Compensation Authority (INAIL). Technical document on the possible reshaping of the containment measures for SARS-CoV-2 infection at the workplaces and prevention strategies. [Documento tecnico sulla possibile rimodulazione delle misure di contenimento del contagio da SARS-Cov-2 nei luoghi di lavoro e strategie di prevenzione]. Ed. INAIL April 2020. ISBN 978-88-7484-911-5
7. Italian Workers' Compensation Authority (INAIL). National Survey of Occupational Safety and Health [Indagine nazionale sulla salute e sicurezza sul lavoro (INSuLa)]. Rome: INAIL, 2014. Available from: https://www.inail.it/cs/internet/docs/allegato_indagine_lavoratori_datori_di_lavoro.pdf. Accessed 2020 July 7
8. Guzzetta G, Poletti P, Ajelli M, Trentini F, Marziano V, Cereda D, et al. Potential short-term outcome of an uncontrolled COVID-19 epidemic in Lombardy, Italy, February to March 2020. Eurosurveillance. 26 Mar 2020;25(12):2000293.
9. Cereda D, Tirani M, Rovida F, Demicheli V, Ajelli M, Poletti P, et al. The early phase of the COVID-19 outbreak in Lombardy, Italy. ArXiv200309320 Q-Bio. 20 Mar 2020; Available at: http://arxiv.org/abs/2003.09320
10. Riccardo F, Ajelli M, Andrianou X, Bella A, Del Manso M, Fabiani M et al. Epidemiological characteristics of COVID-19 cases in Italy and estimates of the reproductive numbers one month into the epidemic. Medrxiv preprint https://doi.org/10.1101/2020.04.08.20056861
11. Zhang J, Litvinova M, Liang Y, Wang Y, Wang W, Zhao S, Wu Q, Merler S, Viboud C, Vespignani A, Ajelli M, Yu H. Changes in contact patterns shape the dynamics of the novel coronavirus disease 2019 outbreak in China. Science, 2020; abb8001.
12. Lavezzo E, Franchin E, Ciavarella C, Cuomo-Dannenburg G, Barzon L, Del Vecchio C, Rossi L, Manganelli R, Loregian A, Navarin N, Abate D. Suppression of a SARS-CoV-2 outbreak in the Italian municipality of Vo'. Nature. 2020 Jun 30:1-5.
13. Pollán M, Pérez-Gómez B, Pastor-Barriuso R, Oteo J, Hernán MA, Pérez-Olmeda M, Sanmartín JL, Fernández-García A, Cruz I, de Larrea NF, Molina M. Prevalence of SARS-CoV-2 in Spain (ENE-COVID): a nationwide, population-based seroepidemiological study. The Lancet. 2020 Jul 6.
14. O'Driscoll M, Ribeiro Dos Santos G, Wang L, Cummings DA, Azman AS, Paireau J, Fontanet A, Cauchemez S, Salje H. Age-specific mortality and immunity patterns of SARS-CoV-2 infection in 45 countries. Nature, 2020
15. Mossong J, Hens N, Jit M, Beutels P, Auranen K, Mikolajczyk R, et al. POLYMOD social contact data (Version 1.1, 2017) [Data set]. Zenodo. http://doi.org/10.5281/zenodo.1059920
16. Italian National Institute of Statistics. Popolazione residente per età, sesso e stato civile al 1 Gennaio 2019. Accessed on March 1st, 2020. Available at: http://demo.istat.it/pop2019/index.html
17. European Agency for Safety and Health at Work (EU-OSHA), 1996. Guidance on risk assessment at work. Available at https://osha.europa.eu/it/legislation/guidelines/guidance-on-risk-assessment-at-work Last access April 22nd 2020
18. U.S. Department of Labor, Employment and Training Administration, 2019. O*NET 24.2 Database.
19. Italian Institute of Statistics (ISTAT), 2009. Classificazione delle attività economiche ATECO 2007 de-rivata dalla Nace Rev. 2. Metodi e Norme n. 40 – 2009.
20. Marinaccio A, Boccuni F, Rondinone BM, Iavicoli S, 2020. Occupational factors in the COVID-19 pandemic in Italy: compensation claims applications support establishing an occupational surveillance system. Occup Environ Med. Epub doi:10.1136/oemed-2020-106844.
21. Poletti P, Tirani M, Cereda D, Trentini F, Guzzetta G, Sabatino G, Marziano V, Castrofino A, Grosso F, Del Castillo G, Piccarreta R. Probability of symptoms and critical disease after SARS-CoV-2 infection. arXiv preprint arXiv:2006.08471. 2020 Jun 15.
22. Trentini F, Marziano V, Guzzetta G, Tirani M, Cereda D, Poletti P, et al. Healthcare strain and intensive care during the COVID-19 outbreak in the Lombardy region: a retrospective observational study on 43,538 hospitalized patients. Medrxiv preprint. https://doi.org/10.1101/2020.11.06.20149690
23. He X, Lau EH, Wu P, Deng X, Wang J, Hao X, Lau YC, Wong JY, Guan Y, Tan X, Mo X. Temporal dynamics in viral shedding and transmissibility of COVID-19. Nature medicine. 2020 May;26(5):672-5.
24. Diekmann O, Heesterbeek JA, Metz JA. On the definition and the computation of the basic reproduction ratio R 0 in models for infectious diseases in heterogeneous populations. Journal of Mathematical Biology. 1990;28(4):365-82.

25. Guzzetta G, Riccardo F, Marziano V, Poletti P, Trentini F, Bella A, Andrianou X, et al. Impact of a nation-wide lockdown on SARS-CoV-2 transmissibility, Italy. Emerging Infectious Diseases, 2020. https://wwwnc.cdc.gov/eid/article/27/1/20-2114_article
26. Okell LC, Verity R, Watson OJ, Mishra S, Walker P, Whittaker C, Katzourakis A, Donnelly CA, Riley S, Ghani AC, Gandy A. Have deaths from COVID-19 in Europe plateaued due to herd immunity?. Lancet (London, England). 2020 Jun 11.
27. WHO Ebola Response Team. Ebola virus disease in West Africa—the first 9 months of the epidemic and forward projections. N Engl J Med. 2014; 371: 1481-1495.
28. Cori A, Ferguson NM, Fraser C, Cauchemez S. A new framework and software to estimate time-varying reproduction numbers during epidemics. American Journal of Epidemiology. 2013;178(9):1505-12.
